# From evidence to implementation: a stakeholder power–interest and social network analysis of potassium-enriched low-sodium salt governance in China

**DOI:** 10.3389/fnut.2026.1910725

**Published:** 2026-09-18

**Authors:** Yuan Shang, Yijin Guo, Chao Gao, Hanning Li, Dongxin Ye, Mei Chen Yap, Ling Wang, Zeng Ge, Nicole Ide, Laura Cobb, Maoyi Tian, Kathy Trieu, Bruce Neal, Xuejun Yin

**Affiliations:** 1 School of Public Health, Institute of Biomedical Innovation, Jiangxi Medical College, Nanchang University, Nanchang, Jiangxi, China; 2 Key Laboratory of Public Nutrition and Health, National Health Commission of the People's Republic of China, National Institute for Nutrition and Health, Chinese Center for Disease Control and Prevention, Beijing, China; 3 Global Health Research Center, Duke Kunshan University, Kunshan, Jiangsu, China; 4 School of Population Medicine and Public Health, Chinese Academy of Medical Sciences & Peking Union Medical College, Beijing, China; 5 Cardiovascular Health Program, Resolve to Save Lives, Vital Strategies (USA) Jinan Representative Office, Jinan, Shandong, China; 6 School of Public Health, Harbin Medical University, Harbin, China; 7 The George Institute for Global Health, University of New South Wales, Sydney, NSW, Australia

**Keywords:** potassium-enriched low-sodium salt, stakeholder mapping, social network analysis, health policy, implementation science, nutrition policy, China

## Abstract

**Objective:**

To characterize the stakeholder landscape and documented governance relationships surrounding the promotion and implementation of potassium-enriched low-sodium salt in China, and to examine institutional and relational patterns relevant to evidence translation.

**Methods:**

We conducted a structured desk review of 115 publicly accessible policy and regulatory documents, industry and implementation materials, and academic and grey-literature sources identified through searches completed in April 2025. Stakeholders were classified by sector and jurisdiction. Two coders independently assessed stakeholder power and interest using predefined documentary criteria and mapped stakeholders into four power–interest categories. Social network analysis was used to examine documented policy/regulatory, technical advisory, collaboration, and advocacy/promotion relationships.

**Results:**

Eighty-three stakeholders were identified across eight categories. Government agencies constituted the largest group (36, 43.4%), followed by professional associations (20, 24.1%) and academic institutions (14, 16.9%). Eight stakeholders (9.6%) were classified as Key Players, 21 (25.3%) as Supporters, 27 (32.5%) as Potential Allies, and 27 (32.5%) as Monitored Stakeholders. Key Players were concentrated among a small number of government, public-health, industry, professional, and media actors, whereas many high-power government bodies had limited documented potassium-enriched low-sodium salt-specific engagement. The four relationship-specific networks contained 25–57 participating stakeholders and had low densities of 0.03–0.10. Policy/regulatory relationships were centered on national and provincial health authorities, while advocacy and technical advisory ties were concentrated around the collective categories of the public and healthcare providers, respectively.

**Conclusion:**

The documented stakeholder landscape was characterized by uneven alignment of power, interest, and institutional connectivity. These patterns present governance challenges for evidence translation but do not establish the causes of limited scale-up. Stronger engagement among high-power actors and clearer coordination across regulatory, technical, industry, and implementation networks are needed to support wider implementation of potassium-enriched low-sodium salt.

## Introduction

Cardiovascular disease has become the leading cause of death worldwide, with elevated blood pressure representing a major modifiable risk factor (1). Excessive sodium intake and insufficient potassium intake are both associated with hypertension and adverse cardiovascular outcomes (2). Reducing sodium intake and increasing potassium intake have been shown to lower blood pressure and mitigate cardiovascular risk (3). Potassium-enriched low-sodium salt, in which part of the sodium chloride is replaced with potassium chloride, offers a practical means of addressing both dietary risks (4). Large-scale randomized trials have demonstrated reductions in blood pressure and cardiovascular events (5), and the World Health Organization issued its first guideline on lower-sodium salt substitutes in 2025, providing recommendations for policymakers, program managers, and health professionals (6). Recent policy and implementation research has increasingly shifted attention from efficacy to the conditions required for population-wide adoption, including regulatory integration, industry engagement, affordability, supply-chain capacity, clinical guidance, and equity (7–9).

China is a particularly important setting because it has generated landmark salt-substitution evidence and issued national guidance on the promotion and use of low-sodium salt, yet large-scale implementation remains uneven. Implementation involves government ministries, regulatory authorities, public-health agencies, professional associations, research institutions, healthcare providers, salt producers and distributors, media organizations, and the public. Evidence from the implementation of other national food-safety standards in China further suggests that gaps between national policy and local practice may arise from uneven interpretation, enforcement, professional capacity, and implementation resources (10).

What remains unclear is not simply which stakeholders participate in potassium-enriched low-sodium salt promotion, but how formal authority and documented engagement are distributed among them; which actors connect evidence generation with regulation, production, professional guidance, and implementation; and where relationships between these functions are weak or absent. Previous stakeholder research has demonstrated the value of analyzing actor positions and networks, but the governance structure surrounding potassium-enriched low-sodium salt implementation in China has not been systematically characterized. We therefore combined document review, power–interest mapping, and social network analysis to identify relevant stakeholders, assess their documented power and interest, and examine policy/regulatory, technical advisory, collaboration, and advocacy/promotion relationships.

## Methods

### Study design

This study employed a policy and stakeholder analysis to examine the institutional landscape surrounding the promotion and use of potassium-enriched low-sodium salt in China. We combined a structured desk review with structured coding of stakeholder attributes and social network analysis. Stakeholder analysis was guided by Mitchell’s stakeholder salience model and Bryson’s framework for public sector stakeholder mapping, both widely applied in health policy research (11, 12). For operational purposes, we prioritized power and interest as the most policy-relevant and directly observable dimensions using documentary evidence. This approach enabled the identification of key actors and the examination of inter-organizational relationships shaping the potassium-enriched low-sodium salt policy environment. Given the national scope and extended time horizon of policies related to potassium-enriched low-sodium salt in China, a document-based design was appropriate for capturing formal institutional roles, regulatory authority, and publicly articulated positions.

### Data sources and document selection

We conducted a structured desk review of publicly accessible materials relevant to the governance, promotion, and implementation of potassium-enriched low-sodium salt in China. Searches were completed in April 2025, with no lower publication-date limit. Three source categories were examined: policy and regulatory documents, industry and implementation materials, and academic and grey literature.

Policy and regulatory sources included the National Health Commission, State Council, Ministry of Industry and Information Technology, State Administration for Market Regulation, China CDC, National Law and Regulation Database, national standards platforms, and provincial and municipal government portals. Industry and implementation sources included official websites and reports of national and subnational salt enterprises, industry associations, the State-owned Assets Supervision and Administration Commission, Xinhua News Agency, and People’s Daily. Academic and grey-literature sources included PubMed, Web of Science, Scopus, CNKI, WanFang, VIP, Google Scholar, study-registration platforms, and the websites of relevant international organizations.

Search terms included the Chinese term “低钠盐,” “富钾低钠盐,” “钾盐,” “代盐,” “盐替代品,” “钠钾比,” “减盐,” and “控盐,” and the English terms “potassium-enriched low-sodium salt,” “low-sodium salt,” “salt substitute,” “reduced-sodium salt,” “sodium reduction,” “implementation,” “promotion,” “policy,” and “stakeholder.” Example search terms are provided in Supplementary Table S2.

Documents were eligible if they explicitly addressed potassium-enriched low-sodium salt or salt substitutes and provided information on policy or regulation, implementation or promotion, stakeholder roles or relationships, or evidence translation. Documents were excluded if they addressed only general salt reduction, lacked a verifiable source, were inaccessible or duplicated, or contained no information relevant to stakeholder identification or relationship mapping. Records were screened independently by two researchers (YS and LW), first against the eligibility criteria and then by full-text review. Duplicate records were checked using title, issuing organization or author, publication year, and source link. Disagreements were resolved through discussion and, when necessary, adjudication by a third researcher (XY).

The search identified 140 records: 66 policy and regulatory records, 41 industry and implementation records, and 33 academic and grey-literature records. During duplicate checking, two duplicate policy/regulatory records were removed, leaving 138 unique records for full-text screening. Following screening, 23 records were excluded—8 policy and regulatory records and 15 academic and grey-literature records—and 115 documents were included. The detailed search and screening procedures are reported in Supplementary Tables S1–S3, and the document-selection process is shown in Supplementary Figure S1.

### Stakeholder identification

Stakeholders were defined as organizations or institutions involved in the promotion, regulation, production, or use of potassium-enriched low-sodium salt in China. Two coders (YS, CG) independently reviewed the included documents to identify relevant stakeholders. Government agencies and departments related to potassium-enriched low-sodium salt were identified from national and sub-national policy documents, regulations, and sodium reduction strategies, while academic institutions, professional associations, and salt producers were identified from academic publications, guidelines, and market reports. Media outlets and non-governmental organizations were identified from media reports, non-governmental organization (NGO) publications, and advocacy materials.

Stakeholder identification followed predefined coding rules and was based on explicit documentary evidence rather than inferred involvement. Full official names were recorded in Chinese and English, and stakeholders were classified as government, industry, academic institutions, professional associations, non-governmental organizations, healthcare providers, media, or the public. The sector definitions and coding rules are provided in Supplementary Table S5.

### Stakeholder profiling

Each identified stakeholder was systematically profiled in terms of power and interest. Power was defined as the capacity to influence decisions concerning potassium-enriched low-sodium salt through formal regulatory authority, agenda-setting responsibility, control over production or distribution, or substantial influence over public communication. Interest was defined as documented potassium-enriched low-sodium salt-specific engagement through policy development, regulation, research, guideline development, implementation, production, promotion, advocacy, or technical support. Power was assessed according to the stakeholder’s institutional authority as of April 2025. Interest was assessed from documented engagement across the included corpus, with the most recent evidence used where roles or activities had changed.

The final power–interest matrix used binary high/low ratings because it was intended as a four-quadrant stakeholder-prioritization tool rather than a continuous measurement scale. High power or high interest required explicit documentary evidence meeting the predefined criteria. For example, an organization with statutory authority over food standards was classified as high power; high interest additionally required documented potassium-enriched low-sodium salt-specific policy or implementation activity, rather than a general reference to sodium reduction alone.

Two coders (YS and CG) independently assigned initial ratings of high, low, or unclear using a standardized coding sheet. “Unclear” was used when the available evidence did not permit confident classification against the predefined criteria and was not treated as low. Initial agreement was 95.2% for power (Cohen’s *κ* = 0.91) and 96.4% for interest (Cohen’s *κ* = 0.92). Unclear and discrepant ratings underwent targeted re-review and consensus adjudication; ratings that remained unresolved were retained as unclear and excluded from quadrant-specific classification.

A sensitivity analysis repeated the power–interest analysis while excluding stakeholders with an initially unclear rating. Stakeholder-level evidence, coding rationales, initial uncertainty status, and final classifications are reported in Supplementary Table S6.

Stakeholders with final high/low ratings were classified as Key Players (high power, high interest), Potential Allies (high power, low interest), Supporters (low power, high interest), or Monitored Stakeholders (low power, low interest).

### Social network analysis

Social network analysis was used to examine documented relationships among stakeholders involved in potassium-enriched low-sodium salt governance and implementation (13). Nodes represented either named stakeholder organizations or collective actor categories, depending on the level of specification in the reviewed documents. “Public” represented population groups targeted by advocacy and promotional activities, while “healthcare providers” represented clinicians and healthcare institutions described collectively in technical advisory, implementation, or collaborative activities. Edges were classified as policy/regulatory, technical advisory, collaboration, or advocacy/promotion relationships. A policy/regulatory relationship was coded when one stakeholder exercised formal authority or oversight over another; a technical advisory relationship when one stakeholder provided expertise or technical guidance to another; a collaboration relationship when stakeholders jointly developed or implemented a policy, program, guideline, or study; and an advocacy/promotion relationship when one stakeholder endorsed, communicated, or promoted potassium-enriched low-sodium salt to another stakeholder or target population. Detailed definitions are provided in Supplementary Table S7.

An edge was recorded only when a document explicitly described a functional relationship between the stakeholders. Co-mention within the same document, without evidence of interaction, authority, advice, joint activity, or promotion, was not sufficient. One document could generate multiple edges when it described multiple distinct relationships. For multi-stakeholder activities, pairwise edges were created only between stakeholders explicitly identified as joint contributors or partners. Repeated mentions of the same stakeholder pair and relationship type within one document were counted once.

Policy/regulatory, technical advisory, and advocacy/promotion relationships were directed according to the documented flow of authority, expertise, or communication; collaboration relationships were undirected. Policy/regulatory edges required an official policy or regulatory source. Other relationship types could be supported by any eligible source that met the same explicit relationship criterion.

Edge weight was defined as the number of independent documents supporting a given stakeholder pair and relationship type. Sources were not otherwise differentially weighted or normalized. Edge weights therefore represent documentary recurrence, not interaction frequency, relationship strength, or causal influence. Relationships documented across the included corpus were aggregated to characterize the network observed up to April 2025; temporal changes in network structure were not analyzed.

Cleaned edge and node tables were imported into Gephi (version 0.10.1) for analysis. Four relationship-specific networks were constructed for policy/regulatory, technical advisory, collaboration, and advocacy/promotion relationships. Each network included stakeholders represented in the corresponding network dataset. Stakeholders from the overall set of 83 who had no documented tie of that relationship type were not included in density or centrality calculations and were reported separately as having no documented ties in that network.

Policy/regulatory, technical advisory, and advocacy/promotion networks were analyzed as directed networks, whereas the collaboration network was analyzed as undirected. Density and centrality were calculated using the full active-node network. Average path length was calculated across all node pairs within the largest weakly connected component of each directed network using its underlying undirected structure and within the largest connected component of the collaboration network. Edge weights were not used in calculating density, average path length, or centrality.

### Reflexivity and bias mitigation

Several authors were affiliated with organizations represented in the stakeholder analysis. To reduce potential interpretive bias, all stakeholder classifications and network relationships were assessed using predefined documentary criteria, independently coded, and resolved through documentary verification and consensus adjudication. Institutional affiliations were not used as evidence in assigning stakeholder classifications or network relationships.

## Results

### Stakeholder distribution and classification

We identified 83 stakeholders across eight categories: government agencies (*n* = 36), professional associations (*n* = 20), academic institutions (*n* = 14), industry (*n* = 6), non-governmental organizations (*n* = 2), media (*n* = 3), healthcare providers (*n* = 1), and the public (*n* = 1). Government agencies accounted for the largest share of stakeholders (43.4%), followed by professional associations (24.1%) and academic institutions (16.9%). Table 1 summarizes stakeholder counts by category and jurisdictional level.

**Table 1:** Distribution of identified stakeholders by sector and level of jurisdiction (*N* = 83).

Stakeholder category	Number	Percentage	Level of Jurisdiction			
			International	National	Provincial	Local
Government	36	43.4%	0	15	11	10
Professional associations	20	24.1%	0	15	0	5
Academic institutions	14	16.9%	1	5	7	1
Industry	6	7.2%	0	3	2	1
non-governmental organizations (NGOs)	2	2.4%	2	0	0	0
Media	3	3.6%	0	2	1	0
Healthcare providers	1	1.2%	0	1	0	0
Public	1	1.2%	0	1	0	0
Total	83	100%	3 (3.6%)	42 (50.6%)	21 (25.3%)	17 (20.5%)

Healthcare providers and the public were represented as nationally aggregated collective actor categories rather than individual organizations.

### Power–interest matrix

Among the 83 stakeholders, 8 (9.6%) were classified as Key Players, 21 (25.3%) as Supporters, 27 (32.5%) as Potential Allies, and 27 (32.5%) as Monitored Stakeholders (Figure 1). Sensitivity analysis excluding the seven stakeholders who required adjudication yielded a similar distribution among the remaining 76 stakeholders: 8 (10.5%) were classified as Key Players, 16 (21.1%) as Supporters, 27 (35.5%) as Potential Allies, and 25 (32.9%) as Monitored Stakeholders. The overall pattern was materially unchanged from the primary analysis.

**Figure 1:**
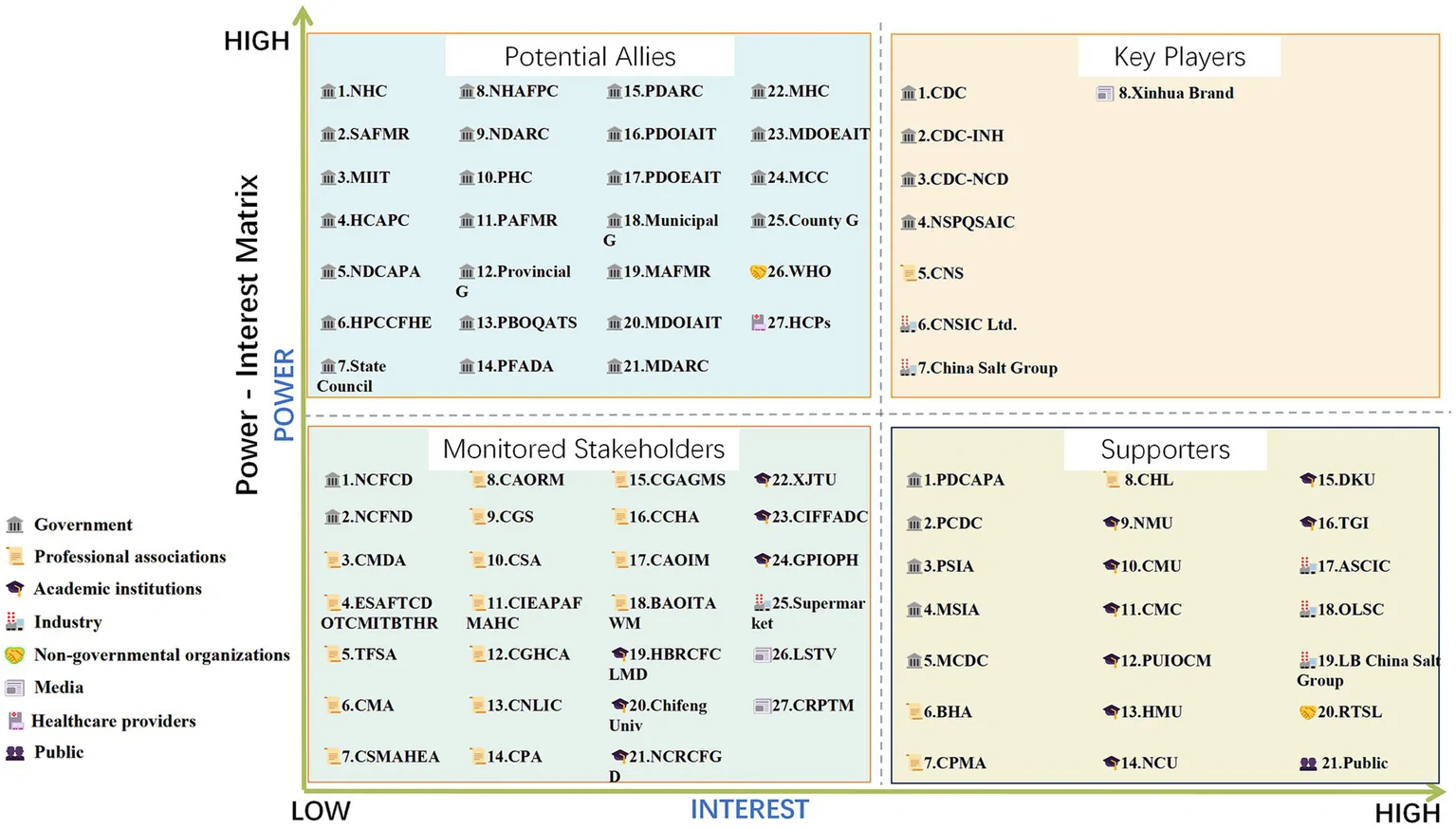
Power–interest matrix of stakeholders involved in potassium-enriched low-sodium salt governance and implementation in China. Stakeholders were classified as Key Players (high power, high interest), Potential Allies (high power, low interest), Supporters (low power, high interest), or Monitored Stakeholders (low power, low interest). Colors indicate stakeholder sectors. “Public” and “healthcare providers” represent collective stakeholder categories. Full stakeholder names and abbreviations are provided in Supplementary Table S8.

Key Players were concentrated among government, public-health, industry, professional, and media actors. China CDC and its affiliated National Institute for Nutrition and Health and National Center for Chronic and Noncommunicable Disease Control and Prevention were classified as high power and high interest based on documented roles in potassium-enriched low-sodium salt guidance, research, and implementation, including the Chinese guideline for promotion and application of potassium-enriched low-sodium salt and national hypertension-prevention activities. The National Salt Product Quality Supervision and Inspection Center was classified as a Key Player because of its documented role in potassium-enriched low-sodium salt standards, product testing, and quality supervision. China National Salt Industry Group and its affiliated entities were similarly classified based on documented production, distribution, and public-promotion activities. Detailed stakeholder-level evidence is provided in Supplementary Tables S6, S9.

Potential Allies were dominated by government bodies with formal regulatory, legal, or supervisory authority but limited documented potassium-enriched low-sodium salt-specific engagement. The National Health Commission, for example, had formal authority over national nutrition and chronic-disease policy, as documented in the *Implementation Plan for the Healthy China Initiative—Cardiovascular and Cerebrovascular Disease Prevention and Treatment Action (2023–2030)* and its response to the proposal on promoting healthy low-sodium salt. However, the reviewed corpus contained comparatively limited evidence of direct and sustained potassium-enriched low-sodium salt-specific implementation activity. Provincial health commissions were classified similarly, although several issued provincial implementation plans incorporating salt reduction and low-sodium salt promotion.

### Stakeholder network analysis

Four stakeholder networks were identified, corresponding to advocacy, collaboration, technical advisory, and policy or regulatory relationships related to potassium-enriched low-sodium salt promotion (Figure 2). The four relationship-specific networks contained two or three components, with the largest components comprising 30 of 32 stakeholders in the policy/regulatory network, 27 of 33 in the technical advisory network, 55 of 57 in the collaboration network, and 21 of 25 in the advocacy/promotion network. Average path lengths within the largest components ranged from 2.15 to 3.06 (Table 2).

**Figure 2:**
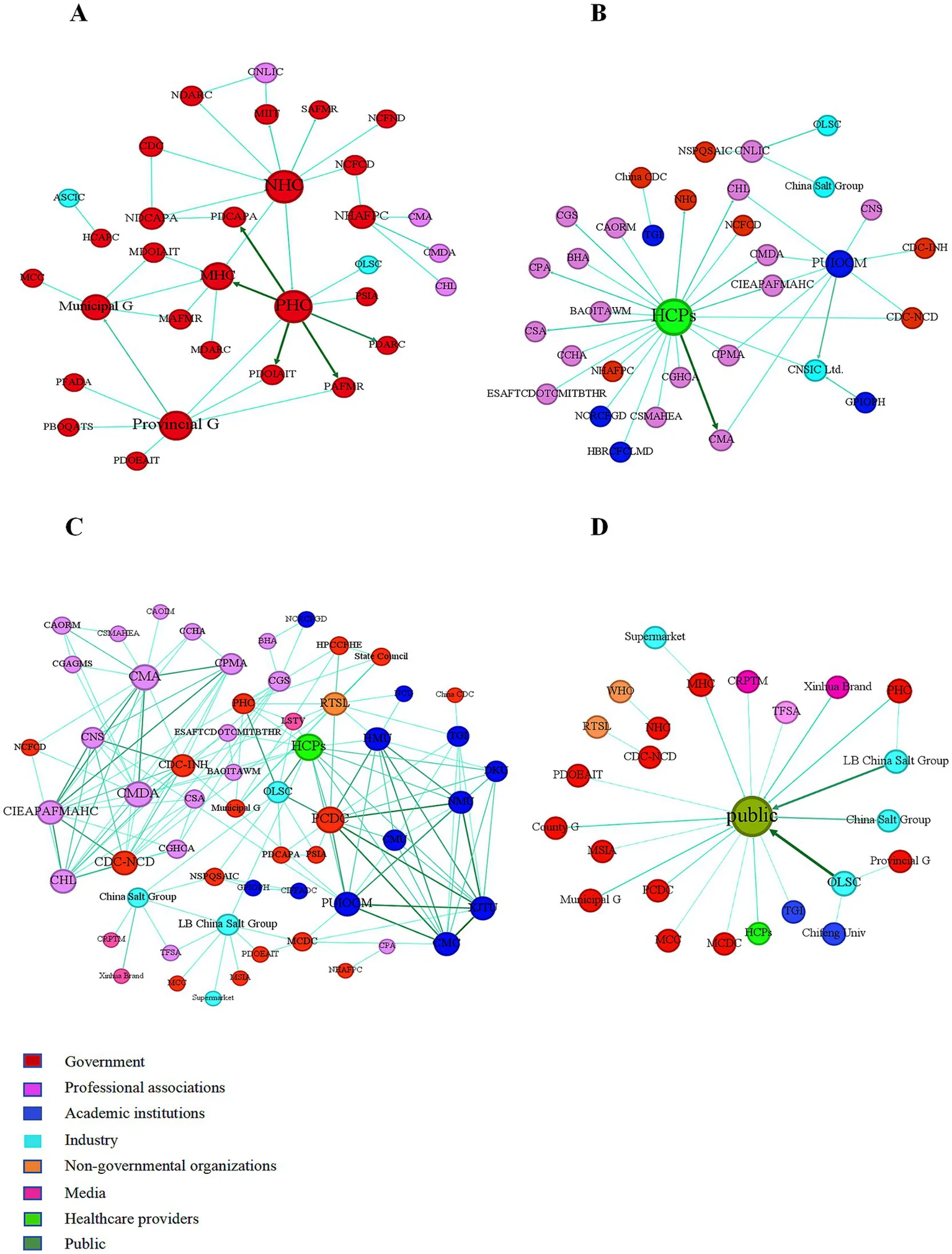
Relationship-specific stakeholder networks for potassium-enriched low-sodium salt governance and implementation in China. **(A)** Policy/regulatory network; **(B)** technical advisory network; **(C)** collaboration network; and **(D)** advocacy/promotion network. Policy/regulatory, technical advisory, and advocacy/promotion relationships are directed, whereas collaboration relationships are undirected. Node color indicates stakeholder sector, node size indicates unweighted degree, and edge width represents the number of independent documents supporting each relationship. Principal central actors are labeled in the main figure; fully labeled enlarged networks are provided in Supplementary Figure S2. Public and healthcare providers represent collective stakeholder categories rather than individual organizations.

**Table 2:** Results of stakeholder network analysis.

Network	Type	Structural characteristics					Cohesion		Centrality		
		Nodes	Edges	Components	Isolates	Largest component nodes	Average path length	Density	Highest in-degrees^†^	Highest out-degrees^‡^	Highest betweenness^§^
Policy/regulatory	Directed	32	39	2	0	30	3.04	0.04	MHC(3)	NHC(9)	PHC(10.5)
Technical advisory	Directed	33	36	3	0	27	2.19	0.03	CNLIC(3) CNSIC Ltd. (3)	HCPs(22)	0
Collaboration	Undirected	57	156	2	0	55	3.06	0.10	N/A	N/A	HCPs(501)
Advocacy/promotion	Directed	25	23	3	0	21	2.15	0.04	Public(17)	OLSC(2) MHC(2) PHC(2)	OLSC(2)

^†^In-degree measures the number of links directed toward a stakeholder, indicating how frequently the stakeholder is referenced, targeted, or receives actions from others within a given network.

^‡^Out-degree measures the number of links initiated by a stakeholder and directed toward others, indicating how frequently the stakeholder provides guidance, initiates actions, or communicates with other actors.

^§^Betweenness centrality values are unnormalized scores and therefore may exceed 1; they represent centrality scores rather than numbers of relationships.

NHC, National Health Commission; PHC, Provincial Health Commission; MHC, Municipal Health Commission; CNLIC, China National Light Industry Council; HCPs, Healthcare providers; OLSC, Other Local Salt Companies; CNSIC Ltd., China National Salt Industry Corporation.

N/A indicates that the measure was not applicable because collaboration relationships were treated as undirected networks and therefore in-degree and out-degree were not calculated. A value of 0 indicates that no stakeholder had a measurable bridging role based on betweenness centrality in the corresponding network. Values in parentheses represent the corresponding centrality scores.

#### Policy/regulatory network

The policy/regulatory network included 32 stakeholders and 39 directed edges. The National Health Commission had the highest out-degree of 9. Municipal health commissions had the highest in-degree highest in-degree of 3, while provincial health commissions had the highest betweenness centrality (10.5). Provincial plans issued in Gansu, Inner Mongolia, Heilongjiang, and Henan provided documentary examples of provincial health commissions translating national priorities into subnational implementation arrangements.

#### Technical advisory network

The technical advisory network included 33 stakeholders and 36 directed edges. Healthcare providers, represented as a collective stakeholder category, had the highest out-degree of 22, indicating that the reviewed documents frequently positioned clinicians and healthcare institutions as providers of dietary and clinical guidance. The China National Light Industry Council and China National Salt Industry Corporation had the highest in-degree of 3, reflecting their documented positions as recipients of technical input associated with low-sodium salt industry standards and industry implementation. Betweenness centrality was zero for all stakeholders in this network.

#### Collaboration network

The collaboration network included 57 stakeholders and 156 undirected edges. Documentary examples included joint guideline development involving public-health institutions and professional associations, and collaborative promotion activities involving China National Salt Industry Group, its local subsidiaries, health authorities, and media organizations. Healthcare providers had the highest betweenness centrality in the primary analysis; because this node represented an aggregated actor category, the value was interpreted as the concentration of documented collaborative relationships involving healthcare providers rather than the influence of a single organization.

#### Advocacy network

The advocacy/promotion network included 25 stakeholders and 23 directed edges. The public had the highest in-degree of 17, indicating that it was the principal collective target of documented promotional activities. Examples included community campaigns organized by China National Salt Industry Group and its provincial or local subsidiaries, as well as joint public-education activities involving the Brand Office of Xinhua News Agency. Other local salt companies had the highest betweenness centrality, reflecting their documented position between promotional organizations and local target populations.

## Discussion

This study provides a comprehensive analysis of the stakeholder landscape shaping the promotion and implementation of potassium-enriched low-sodium salt in China. Despite strong evidence supporting the effectiveness of potassium-enriched low-sodium salt in reducing blood pressure and cardiovascular events (5, 9, 14, 15), our findings reveal a policy environment characterized by a concentration of formal power among a limited number of actors, alongside fragmented engagement across stakeholder groups. Power–interest mapping shows that relatively few stakeholders combine high power with high interest in potassium-enriched low-sodium salt promotion, while social network analysis demonstrates sparse and functionally segmented relationships across advocacy, collaboration, technical advisory, and regulatory domains. These findings identify potential gaps in the documented translation of evidence into coordinated policy and implementation action. They do not establish that governance arrangements caused limited scale-up or exclude other consumer, economic, supply-chain, clinical, market, or regional barriers.

### Power–interest misalignment and limited policy prioritization

A central finding of this study is the persistent misalignment between stakeholder power and engagement with potassium-enriched low-sodium salt. Government agencies and major industry actors possess the greatest decision-making authority over food standards, production, and distribution, yet most government institutions were classified as having high power but low interest in potassium-enriched low-sodium salt. This pattern is reflected in existing national policy documents. For example, in the *Healthy China Initiative—Implementation Plan for the Prevention and Treatment of Cardiovascular and Cerebrovascular Diseases (2023–2030)* (16), potassium-enriched low-sodium salt is mentioned only briefly, with encouragement for producers to manufacture and sell potassium-enriched low-sodium salt, but without dedicated targets, implementation guidance, or regulatory mechanisms. This suggests that potassium-enriched low-sodium salt remains embedded within broader dietary recommendations rather than positioned as a distinct policy priority, despite its strong evidence base. By contrast, many stakeholders demonstrating sustained interest in potassium-enriched low-sodium salt—particularly academic institutions, professional associations, and non-governmental organizations—lack formal authority within regulatory and implementation systems. This distribution suggests a possible gap between evidence generation and the institutional authority required to support coordinated action. Similar patterns have been observed in other nutrition and non-communicable disease policy domains, where interventions that span health, industry, and consumer behavior face diffuse ownership and limited accountability (17, 18).

The positioning of the China National Salt Industry Group and its subsidiaries as Key Players highlights the central role of industry in the potassium-enriched low-sodium salt landscape. Unlike interventions that rely primarily on individual behavior change, potassium-enriched low-sodium salt adoption depends directly on production, formulation, pricing, and distribution decisions. China National Salt Industry Group occupied a distinctive position in the stakeholder landscape because of its national production and distribution capacity and its documented involvement in low-sodium salt promotion. Its role spans commercial supply, market distribution, and public communication, making coordination with health and regulatory authorities particularly relevant to wider implementation. Clear policy direction and regulatory expectations may help align these functions with public-health objectives.

### Fragmented functional networks and weak translation pathways

The social network analysis identified fragmentation across the documented stakeholder networks relevant to potassium-enriched low-sodium salt implementation. Advocacy, regulatory, technical advisory, and collaborative functions are distributed across largely separate networks, with limited overlap and few bridging actors. Advocacy activities involve a range of organizations, with local salt enterprises and regional branches of China National Salt Industry Group playing prominent roles in public communication. However, coordination across advocacy actors is limited, and professional associations and academic institutions are only weakly connected within this network. By contrast, the policy/regulatory network is highly centralized and dominated by government actors, with clear vertical transmission from national to local levels. While this structure supports administrative coherence, it is only weakly connected to technical advisory and advocacy networks. Regulatory documents contained limited explicit attention to potassium-enriched low-sodium salt as a distinct policy issue, consistent with its low documented prioritization among several high-power actors. The technical advisory network is characterized by short, direct connections between healthcare providers, academic institutions, professional associations, and industry actors, indicating concentrated technical capacity. However, the low density indicates that documented technical advisory relationships were concentrated among a limited number of institutions. Clear documented pathways linking research evidence with regulatory action were not consistently identified. Although the collaboration network included a broad range of stakeholders, documented relationships were loosely connected and organized around a small number of bridging actors.

### Implications for multi-sectoral policy action

Taken together, these findings highlight a governance configuration in which potassium-enriched low-sodium salt promotion relies on fragmented advocacy, centralized but weakly engaged regulation, technically strong yet siloed advisory capacity, and collaboration dependent on a small number of core actors. This configuration mirrors challenges observed in other public health domains, including tobacco control and salt reduction initiatives in high-income countries, where early progress often depended on sustained advocacy by academic and civil society actors before stronger government stewardship emerged (19, 20). International experience suggests that clearer institutional leadership and multi-sector coordination can facilitate implementation of food system interventions (21, 22). For example, Iran’s regulation of edible oil reformulation was coordinated by a central food safety authority that integrated evidence and stakeholder input into a coherent regulatory framework (23). Similarly, in the United Kingdom, early advocacy by academia and civil society preceded more comprehensive government-led salt reduction policies (19). These examples do not prescribe a specific pathway for China, but they illustrate how alignment between evidence, authority, and coordination can support population-level dietary interventions. Thus, accelerating the scaled implementation of potassium-enriched low-sodium salt in China may be achieved by mobilizing high-influence stakeholders and prioritizing its promotion as an explicit policy priority, strengthening cross-network collaboration and establishing regular multi-stakeholder platforms to break down silos between academia, salt producers, and regulatory agencies while creating effective pathways for translating research evidence into regulatory action, and aligning regulatory oversight and industry incentives.

### Strengths and limitations

This study extends existing research on potassium-enriched low-sodium salt by moving beyond effectiveness evidence to examine the institutional and relational conditions shaping implementation. By integrating stakeholder power–interest analysis with social network analysis, we provide a structured approach to understanding how governance arrangements, market structures, and inter-organizational relationships influence the translation of evidence into policy and practice. This approach may be relevant to other settings where nutrition and NCD interventions depend on coordinated action across multiple sectors. A key strength of this study is the comprehensive analysis of publicly available policy documents and organizational records across an extended policy period, allowing systematic assessment of formal stakeholder roles and documented relationships. However, reliance on documentary sources alone may underrepresent informal influence, negotiation, and unpublished interactions. In addition, the binary operationalization of power and interest enhances transparency but may oversimplify gradients of influence and engagement. Several authors are affiliated with organizations represented in the stakeholder analysis, which may introduce potential interpretive bias despite the use of predefined coding criteria, independent coding, and consensus adjudication. Future research incorporating qualitative interviews or participatory methods could further elucidate these dynamics.

## Conclusion

The documented governance landscape for potassium-enriched low-sodium salt in China was characterized by uneven alignment of stakeholder power, interest, and institutional connectivity. These patterns may present governance challenges for evidence translation and wider implementation. However, the analysis relied on publicly available documents, which may underrepresent informal or unpublished stakeholder roles and relationships, and the binary power–interest classification necessarily simplifies variation in stakeholder influence and engagement. The findings should therefore be interpreted as a structured description of the documented policy environment rather than a complete or causal explanation of limited scale-up. Stronger engagement among high-power actors and clearer coordination across regulatory, technical, industry, and implementation networks may support broader implementation.

## Data Availability

The original contributions presented in the study are included in the article/Supplementary material, further inquiries can be directed to the corresponding author/s.
