# Supplementary material for: From evidence to implementation: a stakeholder power–interest and social network analysis of potassium-enriched low-sodium salt governance in China
Source: Front Nutr. 2026 Sep 18;13:1910725. doi: 10.3389/fnut.2026.1910725 (PMC13630623; doi:10.3389/fnut.2026.1910725)
Supplement: Supplementary file 1 [file Supplementary_file_1.DOCX]

**Supplementary Materials**

Table of Contents

[Table S1 Document categories, subtypes, and analytic roles	2](#_Toc7857)

[Table S2 Search strategy and selection criteria by document category	4](#_Toc9509)

[Table S3 Document screening process	6](#_Toc31056)

[Table S4 List of Included Documents.	7](#_Toc31470)

[Table S5 Stakeholder Identification and Coding Framework.	26](#_Toc7588)

[Table S6 Evidence supporting stakeholder power and interest classification, including documentary sources, coding rationale, uncertainty status, and final decisions after review.	28](#_Toc6399)

[Table S7 Definitions of relationship types.	52](#_Toc11749)

[Table S8 Full Names For All Stakeholder Abbreviations	53](#_Toc1534)

[Table S9 Documentary evidence supporting the classification and network positions of the eight Key Players	57](#_Toc30276)

[Supplementary Figure S1.	59](#_Toc17649)

[Supplementary Figure S2.	60](#_Toc7902)

**Table S1 Document categories, subtypes, and analytic roles**

| Document category | Document subtype | Primary analytic purpose | Key inclusion criteria | Role in stakeholder analysis |
| --- | --- | --- | --- | --- |
| Policy and regulatory documents | Policy, Action Plan / Strategy Document, Program / Initiative | Identify formal authority, governance priorities, and implementation pathways | Officially issued or endorsed government documents that explicitly mention potassium-enriched low-sodium salt, low-sodium salt, potassium-enriched salt, or potassium-containing salt substitutes as part of policy direction, strategic priorities, or government-led implementation activities. | Identification of government stakeholders, policy actors, and implementation agencies |
|  | Guideline | Characterize official technical recommendations and advisory mechanisms | Officially issued or endorsed technical guidance that explicitly provides recommendations, cautions, indications, contraindications, or implementation advice related to potassium-enriched low-sodium salt or potassium-containing salt substitutes. | Identification of technical advisory stakeholders |
|  | Standard | Characterize technical and regulatory requirements | National or subnational standards that explicitly address potassium-enriched low-sodium salt, low-sodium salt, or potassium-containing salt substitutes in relation to production, composition, labeling, safety, or quality requirements | Identification of regulatory and technical stakeholders |
| Industry and implementation materials | Industry initiative | Capture real-world promotion activities and implementation practices | Publicly verifiable industry-led or industry–government collaborative activities that explicitly promote, distribute, introduce, or educate the public about potassium-enriched low-sodium salt or potassium-containing salt substitutes | Identification of industry stakeholders and implementation networks |
|  | Public Education Resource | Document communication and advocacy activities | Publicly available educational materials or communication resources that explicitly refer to potassium-enriched low-sodium salt, low-sodium salt, potassium-enriched salt, or potassium-containing salt substitutes, rather than general salt reduction alone | Mapping advocacy and public engagement relationships |
| Academic and grey literature | Academic Literature | Contextualize scientific evidence and identify academic stakeholders | Peer-reviewed studies explicitly focused on potassium-enriched low-sodium salt, low-sodium salt, potassium-enriched salt, or potassium-containing salt substitutes, including evidence on effectiveness, safety, acceptability, utilization, implementation, or scale-up among Chinese populations | Identification of academic stakeholders and interpretation of evidence translation |
|  | Study Registration Platform | Identify ongoing or registered research activities | Clinical trial registrations or study platform records explicitly evaluating potassium-enriched low-sodium salt, low-sodium salt, potassium-enriched salt, or potassium-containing salt substitutes | Identification of research stakeholders and evidence generation activities |
|  | International reports | Contextualize global policy environment and implementation approaches | Reports, guidelines, policy briefs, or implementation frameworks from international organizations that explicitly discuss potassium-enriched low-sodium salt, lower-sodium salt substitutes, potassium-enriched salt, or potassium-containing salt substitutes | Interpretation of policy–evidence alignment and identification of international stakeholders |

**Table S2 Search strategy and selection criteria by document category**

| Item | Policy and regulatory documents | Industry and implementation materials | Academic and grey literature |
| --- | --- | --- | --- |
| Objective | To identify national and subnational policies, regulations, standards, guidelines, and government-led initiatives related to governance, promotion, and implementation of potassium-enriched low-sodium salt in China | To identify industry stakeholders, promotion activities, and implementation practices related to potassium-enriched low-sodium salt beyond formal policy documents | To identify China-based empirical evidence and international guidance documents relevant to potassium-enriched low-sodium salt policy development, implementation, and scale-up |
| Review period | Up to April 2025; no lower publication-date limit | Up to April 2025; no lower publication-date limit | Up to April 2025; no lower publication-date limit |
| Data sources | National Health Commission; State Council; Ministry of Industry and Information Technology; State Administration for Market Regulation; Chinese Center for Disease Control and Prevention; National Law and Regulation Database; provincial and municipal government portals; CNKI; WanFang Database; VIP Information | Official websites and activity reports of national and provincial salt enterprises; government portals; State-owned Assets Supervision and Administration Commission; official media platforms including Xinhua and People’s Daily | PubMed; Web of Science; Scopus; CNKI; WanFang Database; Google Scholar; international organization websites including WHO, Resolve to Save Lives, and The George Institute for Global Health |
| Search strategy | Broad policy-related terms (e.g., “三减三健”, “减盐行动”, “慢性病防控”, “健康中国行动”) were used for initial identification of relevant policy documents, and records were retained only if they contained explicit references to potassium-enriched low-sodium salt or salt substitutes. Example search string: (“potassium-enriched low-sodium salt” OR “low-sodium salt” OR “salt substitute” OR “reduced-sodium salt” OR “低钠盐” OR “富钾低钠盐” OR “盐替代品”) AND (“policy” OR “regulation” OR “guideline” OR “standard” OR “strategy” OR “三减三健” OR “减盐行动”). | Keyword-based searching focused on publicly available industry materials and implementation activities related to potassium-enriched low-sodium salt or salt substitutes, supplemented by snowball searching through relevant references and hyperlinks. | Database-specific searches used reproducible combinations of salt substitute-related terms, China-related terms, and implementation or policy-related terms. Reference lists of included studies and relevant reports were manually screened. |
| Search terms | (“potassium-enriched low-sodium salt” OR “low-sodium salt” OR “salt substitute” OR “reduced-sodium salt” OR “低钠盐” OR “富钾低钠盐”) AND (policy OR regulation OR guideline OR standard OR strategy OR implementation) | (“potassium-enriched low-sodium salt” OR “low-sodium salt” OR “salt substitute” OR “reduced-sodium salt” OR “低钠盐” OR “富钾低钠盐”) AND (promotion OR implementation OR education OR campaign OR program OR distribution) | (“potassium-enriched low-sodium salt” OR “low-sodium salt” OR “salt substitute” OR “reduced-sodium salt” OR “低钠盐” OR “富钾低钠盐”) AND (China OR Chinese) AND (implementation OR acceptability OR utilization OR policy OR scale-up OR stakeholder) |
| Inclusion criteria | Officially issued or endorsed documents that were publicly accessible and explicitly referenced potassium-enriched low-sodium salt or related implementation strategies | Publicly verifiable industry-led or collaborative activities involving promotion, education, or implementation of potassium-enriched low-sodium salt | Peer-reviewed studies conducted in China evaluating effectiveness, safety, acceptability, utilization, implementation, or scale-up; international guidelines, reports, or implementation frameworks relevant to potassium-enriched low-sodium salt |
| Exclusion criteria | Documents focusing only on general salt reduction without explicit potassium-enriched low-sodium salt reference; non-official documents without policy relevance; documents lacking source transparency | Pure commercial advertising, sales promotion without public health relevance, or unverifiable materials; activities unrelated to potassium-enriched low-sodium salt or salt substitutes | Studies without China-specific evidence or implementation relevance; studies focusing only on general sodium reduction; documents unrelated to potassium-enriched low-sodium salt; inaccessible documents |

**Table S3 Document screening process**

| Document category | Records identified | Duplicate records removed | Full-text reviewed | Excluded after full-text screening | Reasons for exclusion | Included documents |
| --- | --- | --- | --- | --- | --- | --- |
| Policy and regulatory documents | 66 | **2** | 64 | 8 | Documents did not explicitly mention potassium-enriched low-sodium salt, low-sodium salt, or relevant potassium-containing salt substitutes. | 56 |
| Industry and implementation materials | 41 | 0 | 41 | 0 | None | 41 |
| Academic and grey literature | 33 | 0 | 33 | 15 | Did not meet inclusion criteria, including lack of China-specific evidence, insufficient implementation or policy relevance, or no direct relevance to potassium-enriched low-sodium salt | 18 |
| Total | 140 | **2** | 138 | 23 | — | 115 |

Note: A total of 140 records were initially identified. During duplicate checking, two duplicate records were identified within the policy and regulatory document category and removed, leaving 138 unique records for full-text screening. Following full-text assessment, 23 records were excluded, resulting in 115 documents included in the final analysis.

**Table S4 List of Included Documents.**

| Document ID | Document title | Year of publication | Document category | Source / affiliated organization(s) | Explicit reference to potassium-enriched low-sodium salt or salt substitutes |
| --- | --- | --- | --- | --- | --- |
| 1 | WHO's Salt Substitution Guidelines for Population-Wide Impact: Act on Strong Evidence, Monitor for the Long Term. | 2025 | Academic Literature | World Health Organization；Elsevier BV | Yes |
| 2 | To evaluate the effect of low-sodium salt on systolic blood pressure in patients with stage G2 and G3a chronic kidney disease | 2025 | Study Registration Platform | School of Public Health, Harbin Medical University；Chinese Clinical Trial Registry | Yes |
| 3 | Use of Lower-Sodium Salt Substitutes: WHO Guideline | 2025 | Guideline | World Health Organization | Yes |
| 4 | Effect of salt substitution on fracture - a secondary analysis of the Salt Substitute and Stroke Study (SSaSS). | 2024 | Academic Literature | Ningxia Medical University；BioMed Central Ltd. | Yes |
| 5 | The best of 'best buys': public health values of potassium-enriched salt substitute. | 2024 | Academic Literature | Peking University Institute of Clinical Medicine；Taylor & Francis Ltd.（UK） | Yes |
| 6 | Cost-Effectiveness of Salt Substitute and Salt Supply Restriction in Eldercare Facilities: The DECIDE-Salt Cluster Randomized Clinical Trial. | 2024 | Academic Literature | Peking University Institute of Clinical Medicine；American Medical Association | Yes |
| 7 | Saving Lives Through Comprehensive Sodium Reduction: A 6-Step Guide | 2024 | Guideline | Resolve to Save Lives | Yes |
| 8 | Scaling Up the Use of Potassium-Enriched Salt | 2024 | Academic Literature | The George Institute for Global Health | Yes |
| 9 | The availability, price, and characteristics of low sodium salt based on an online salt market survey in China: Implications for scaling up its use. | 2023 | Academic Literature | Health Promotion Department, Chinese Center for Health Education；Wiley-Blackwell Publishing Ltd | Yes |
| 10 | National Initiatives on Salt Substitutes: Scoping Review. | 2023 | Academic Literature | National Institute for Nutrition and Health, Chinese Center for Disease Control and Prevention；JMIR Publications Inc. | Yes |
| 11 | Sodium Reduction Framework | 2023 | Action Plan/Strategy Document | Resolve to Save Lives | Yes |
| 12 | Preventing Cardiovascular Disease Through Salt Reduction | 2023 | Policy | Resolve to Save Lives | Yes |
| 13 | Why Do We Need to #SwitchTheSalt? | 2023 | Public Education Resource | The George Institute for Global Health | Yes |
| 14 | Cost-Effectiveness of a Household Salt Substitution Intervention: Findings From 20 995 Participants of the Salt Substitute and Stroke Study. | 2022 | Academic Literature | School of Public Health, Harbin Medical University；Lippincott Williams & Wilkins | Yes |
| 15 | Low-Sodium Salt Pilot Promotion Project in Heilongjiang | 2022 | Study Registration Platform | School of Public Health, Harbin Medical University；Chinese Clinical Trial Registry | Yes |
| 16 | Protocol for the economic evaluation of the China Salt Substitute and Stroke Study (SSaSS). | 2021 | Academic Literature | Department of Cardiology, Peking University Third Hospital；BMJ Publishing Group Ltd. | Yes |
| 17 | Factors Associated With the Use of a Salt Substitute in Rural China. | 2021 | Academic Literature | Peking University Clinical Research Institute；American Medical Association | Yes |
| 18 | Policy Brief: Salt Substitutes | 2021 | Policy | The George Institute for Global Health | Yes |
| 19 | Integrated Traditional Chinese and Western Medicine Diagnosis and Treatment Guidelines for Diabetes Complicated with Hypertension | 2025 | Guideline | Chinese Medical Doctor Association Integrative Medicine Branch Endocrinology and Metabolic Diseases Committee | Yes |
| 20 | Chinese Diagnosis and Treatment Guidelines for Obesity (2024 Edition) | 2025 | Guideline | National Health Commission of the People's Republic of China Medical Administration Department | Yes |
| 21 | 2024 National Hypertension Day: Healthy Weight, Ideal Blood Pressure | 2024 | Program/Initiative | National Health Commission Office | Yes |
| 22 | Chinese Hypertension Clinical Practice Guidelines | 2024 | Guideline | Chinese Medical Association Cardiovascular Disease Branch, Chinese Medical Association | Yes |
| 23 | Hypertension Nutrition and Exercise Guidance Principles (2024 Edition) Guidelines | 2024 | Guideline | National Health Commission Office | Yes |
| 24 | Chinese Guideline for Promotion and Application of Low-Sodium Salt(2024) | 2024 | Guideline | Chinese Preventive Medicine Association Health Risk Assessment and Control Professional Committee, Chinese Preventive Medicine Association | Yes |
| 25 | Chinese Food Industry Salt Reduction Guideline | 2024 | Guideline | National Institute for Nutrition and Health, Chinese Center for Disease Control and Prevention, Chinese Nutrition Society | Yes |
| 26 | Chinese Guidelines for Prevention and Treatment of Hypertension (2024 Revision) | 2024 | Guideline | Chinese Hypertension Prevention and Treatment Guideline Revision Committee, Chinese Hypertension Journal | Yes |
| 27 | Chinese Clinical Diagnosis and Treatment Guidelines for Hypertension in Renal Transplant Recipients (2024) | 2024 | Guideline | Chinese Medical Association Organ Transplantation Society | Yes |
| 28 | Guidelines for Rational Drug Use and Comprehensive Management in County-level Medical Institutions | 2024 | Guideline | Chinese Pharmacists Association | Yes |
| 29 | Notice on Issuing the Implementation Plan for the Healthy China Initiative - Cardiovascular and Cerebrovascular Diseases Prevention and Treatment Action (2023-2030) | 2023 | Action Plan / Strategy Document | National Health Commission, National Development and Reform Commission, Ministry of Education, Ministry of Science and Technology, Ministry of Industry and Information Technology, Ministry of Civil Affairs, Ministry of Finance, State Administration for Market Regulation, General Administration of Radio and Television, State General Administration of Sport, National Administration of Traditional Chinese Medicine, National Center for Disease Control and Prevention, All-China Federation of Trade Unions, China Red Cross Society | Yes |
| 30 | Adult Hypertension Dietary Guidelines (2023 Edition) | 2023 | Guideline | National Health Commission Office | Yes |
| 31 | Chinese Guidelines for Management of Hypertension in the Elderly 2023 | 2023 | Guideline | Chinese Geriatrics Society Hypertension Branch, Chinese Hypertension Journal | Yes |
| 32 | Guidelines for Preventive Intervention in Pre-hypertension (Treating Disease Before Onset) | 2023 | Guideline | Writing Group for 'Preventive Intervention Guidelines for Hypertension Disease', Beijing University of Chinese Medicine | Yes |
| 33 | Chinese Guidelines for Primary Prevention of Cardiovascular Diseases (Grassroots Edition) | 2023 | Guideline | Chinese Medical Association Cardiovascular Disease Society; Chinese Medical Association | Yes |
| 34 | Chinese Stroke Association Guidelines for Clinical Management of Cerebrovascular Diseases (Second Edition) (Excerpt) — Chapter Four: Clinical Management Recommendations for Ischemic Cerebrovascular Diseases | 2023 | Guideline | Chinese Stroke Society Chinese Clinical Management Guidelines for Cerebrovascular Disease Writing Committee, Chinese Stroke Society | Yes |
| 35 | Chinese Guidelines for Management of Hypertension in Patients with Chronic Kidney Disease (2023 Edition) | 2023 | Guideline | Chinese Society of Nephrology | Yes |
| 36 | Healthy China Salt Reduction Action \| China Salt Group Collaborates with China State Railway Group to Care for National Health | 2023 | Industry initiative | China National Salt Industry Group Co., Ltd | Yes |
| 37 | Chinese Dietary Guidelines (2022) | 2022 | Guideline | Chinese Nutrition Society | Yes |
| 38 | Chinese Guidelines for Secondary Prevention of Ischemic Stroke and Transient Ischemic Attack 2022 | 2022 | Guideline | Chinese Medical Association Neurology Branch, Chinese Medical Association Neurology Branch Cerebrovascular Disease Group | Yes |
| 39 | Reply to Proposal No. 5372 of the Third Session of the 13th National People's Congress "Suggestions on Establishing the Greater Health Concept, Scientific Salt Consumption, and Vigorously Promoting Healthy Low-Sodium Salt" | 2021 | Policy | National Health Commission | Yes |
| 40 | Chinese clinical practice guidelines for nutritional treatment of chronic kidney disease (2021) | 2021 | Guideline | Chinese Medical Doctor Association Renal Physicians Branch, Chinese Medical Doctor Association | Yes |
| 41 | Light Industry Standard of the People's Republic of China QB/T 2019-2020 Low-Sodium Salt | 2020 | Standard | Ministry of Industry and Information Technology of the People's Republic of China | Yes |
| 42 | Explanation for the Draft of GB2721 "National Food Safety Standard — Edible Salt" | 2020 | Standard | Guangdong Provincial Institute of Public Health | Yes |
| 43 | Chinese Guidelines for a Healthy Lifestyle to Prevent Cardiometabolic Diseases (2020) | 2020 | Guideline | Chinese Preventive Medicine Association | Yes |
| 44 | The Healthy China Initiative (2019-2030) | 2019 | Policy | Healthy China Action Promotion Committee | Yes |
| 45 | Notice of the Disease Control Bureau of the National Health Commission on Carrying out the 2019 National Healthy Lifestyle Month Campaign — Sep 15 Salt Reduction Awareness Week | 2019 | Program/Initiative | National Health Commission Disease Control Bureau | Yes |
| 46 | Chinese Guidelines for Cardiovascular Disease Risk Assessment and Management | 2019 | Guideline | Joint Committee for Writing Chinese Cardiovascular Disease Risk Assessment and Management Guidelines, Chinese Circulation Journal | Yes |
| 47 | Guidelines for Rational Drug Use in Heart Failure (Second Edition) | 2019 | Guideline | National Health and Family Planning Commission Rational Drug Use Expert Committee, China Pharmacist Association | Yes |
| 48 | Guidelines for Comprehensive Prevention and Control of Cardiovascular Diseases in Community Populations (Trial) | 2017 | Guideline | Community Population Cardiovascular Disease Comprehensive Prevention and Control Guidelines (Trial) Editorial Board, Chinese Medical Doctor Association | Yes |
| 49 | Guidelines for Rational Drug Use in Hypertension (Second Edition) | 2017 | Guideline | National Health and Family Planning Commission Rational Drug Use Expert Committee, China Medical Association | Yes |
| 50 | GB2721-2015 National Food Safety Standard — Edible Salt | 2016 | Standard | National Health and Family Planning Commission | Yes |
| 51 | Chinese Hypertension Patient Education Guidelines | 2013 | Guideline | Hypertension Alliance (China), Chinese Medical Association | Yes |
| 52 | QB/T 2019-2005 (Recommended) | 2005 | Standard | National Development and Reform Commission of the People's Republic of China | Yes |
| 53 | QB 2019-1994 (Mandatory) | 1994 | Standard | China National Light Industry Council | Yes |
| 54 | China Salt Heilongjiang Company Organizes "Inheriting the Spirit of Lei Feng, Advocating Healthy Salt Consumption - Low-Sodium Salt Science Popularization Activity" | 2025 | Industry initiative | China National Salt Industry Group Heilongjiang Salt Industry Co., Ltd. | Yes |
| 55 | Salt Reduction, Not Saltiness Reduction, For Shared Health — Qiannan Company Promotes Low-Sodium Salt Science Education in Communities and Schools | 2025 | Industry initiative | Guizhou Salt Industry Group Qiannan Company | Yes |
| 56 | Qianxinan Company Conducts Low-Sodium Salt Publicity and Science Education Activities | 2025 | Industry initiative | Guizhou Salt Industry Group Qiannan Company | Yes |
| 57 | Sanming Salt Industry Conducts Low-Sodium Salt Themed Promotion Activities at the Traditional Chinese Medicine Night Market | 2025 | Industry initiative | Fujian Sanming Salt Industry Co., Ltd. | Yes |
| 58 | Guizhou Salt Group's Grassroots Units Carry Out Low-Sodium Salt Publicity and Promotion to Facilitate the Effective Implementation of the Healthy China Action | 2025 | Industry initiative | Guizhou Salt Industry Group Co., Ltd | Yes |
| 59 | Healthy Gansu Action — Implementation Plan for Cardiovascular and Cerebrovascular Disease Prevention and Treatment Action (2024–2030) | 2024 | Action Plan / Strategy Document | Gansu Provincial Health Commission | Yes |
| 60 | Healthy Inner Mongolia Action — Implementation Plan for Cardiovascular and Cerebrovascular Disease Prevention and Treatment Action (2024–2030) | 2024 | Action Plan / Strategy Document | Inner Mongolia Autonomous Region Health Commission | Yes |
| 61 | Healthy Longjiang Action — Implementation Plan for Cardiovascular and Cerebrovascular Disease Prevention and Treatment Action (2024–2030) | 2024 | Action Plan / Strategy Document | Heilongjiang Provincial Health Commission | Yes |
| 62 | Healthy Henan Action — Implementation Plan for Cardiovascular and Cerebrovascular Disease Prevention and Treatment Action (2024–2030) | 2024 | Action Plan / Strategy Document | Henan Provincial Health Commission | Yes |
| 63 | National Hypertension Day｜Healthy China, Salt Reduction Action - China Salt Fujian in Action | 2024 | Industry initiative | China National Salt Industry Group Fujian Salt Industry Co., Ltd. | Yes |
| 64 | Notice on Carrying Out the Municipal "Greeting the 20th Party Congress, National Day, and CIIE" 100-Day Patriotic Health Campaign | 2022 | Program/Initiative | Shanghai Municipal Health Commission | Yes |
| 65 | Healthy Inner Mongolia Rational Diet Action Implementation Plan | 2020 | Action Plan / Strategy Document | Inner Mongolia Autonomous Region Health Commission | Yes |
| 66 | Notice of the Healthy Guangxi Action Promotion Committee on Issuing the Healthy Guangxi Action (2020–2030) | 2020 | Policy | Healthy Guangxi Action Promotion Committee | Yes |
| 67 | Heilongjiang Province Rational Diet Action Provincial-Ministry Cooperation Project Implementation Plan | 2020 | Action Plan / Strategy Document | Heilongjiang Provincial People's Government Office | Yes |
| 68 | Implementation Opinions of the Autonomous Region People's Government on Healthy Ningxia Action | 2019 | Action Plan / Strategy Document | Ningxia Hui Autonomous Region People's Government | Yes |
| 69 | Notice of the General Office of the Sichuan Provincial People's Government on Printing and Distributing the Implementation Plan for Strengthening and Expanding the Sichuan Salt Industry (Repealed) | 2017 | Action Plan / Strategy Document | Sichuan Provincial People's Government Office | Yes |
| 70 | Opinions of the Provincial Government on Deepening the Construction of "Healthy Jiangsu" and Continuously Improving the Health Standards of the People | 2015 | Action Plan / Strategy Document | Jiangsu Provincial People's Government | Yes |
| 71 | Notice on the Working Rules and Division of Responsibilities of Member Units of the Shandong Provincial Patriotic Health Campaign Committee | 2014 | Action Plan / Strategy Document | Shandong Provincial Patriotic Health Campaign Committee | Yes |
| 72 | Zhong'an Online: Anhui Launches "Low-Salt Diet for Health" Campaign to Promote the Use of Low-Sodium Salt | 2014 | Program/Initiative | Anhui State-owned Assets Supervision and Administration Commission | Yes |
| 73 | Beijing Launches "Salt Limitation Action" to Promote Low-Sodium Salt in 28 Chain Supermarkets | 2011 | Program/Initiative | Beijing Municipal Health Bureau | Yes |
| 74 | Healthy Beijinger — Ten-Year Action Plan for National Health Promotion | 2009 | Action Plan / Strategy Document | Beijing Municipal Government | Yes |
| 75 | China Salt Shanghai Company Conducts Theme Activities for "Iodine Deficiency Disorders Prevention Day" | 2025 | Industry initiative | China National Salt Industry Group Shanghai Salt Industry Co., Ltd. | Yes |
| 76 | Launching a Healthy Revolution in the Kitchen, Unlocking the Code of Taste — China Salt Heilongjiang Salt Industry Group Co., Ltd. Conducts Public Welfare Lecture and Community Activities on "Salt Reduction and Salt Replacement, Low Sodium for Health" | 2025 | Industry initiative | China National Salt Industry Group Heilongjiang Salt Industry Co., Ltd. | Yes |
| 77 | China Salt (Shanxi) Salt Industry Co., Ltd. Conducts "Vibrant Spring, Most Beautiful 'Salt' Value" Healthy Salt Replacement Activity | 2025 | Industry initiative | China National Salt Industry Group Shanxi Salt Industry Co., Ltd. | Yes |
| 78 | Tongren Company Implements Multiple Measures to Carry Out Low-Sodium Salt Publicity and Promotion | 2025 | Industry initiative | Guizhou Salt Industry Group Tongren Company | Yes |
| 79 | Fragrant Rice Dumplings for Dragon Boat Festival, "Salt" Protects Health — Qiannan Company Conducts Low-Sodium Salt Publicity and Promotion | 2025 | Industry initiative | Duyun City Guanghui Community | Yes |
| 80 | Bijie Company Qianxi Branch Brings Low-Sodium Salt Publicity to the Guizhou Beef Noodle Cultural Tourism Season Event Site | 2025 | Industry initiative | Guizhou Salt Industry Group Bijie Company Qianxi Branch | Yes |
| 81 | Hunan Salt Industry Group Awarded "Low-Sodium Salt, Ecological Salt Promotion Demonstration Unit" | 2025 | Industry initiative | Hunan Salt Industry Group Co., Ltd. | Yes |
| 82 | The Second Party Branch of China Salt Exploration Institute Jointly with the Youth League Branch Conducts "Healthy China Tour - China Salt Low-Sodium Salt Enters the Community" Activity | 2024 | Industry initiative | China Salt Survey and Design Institute Co., Ltd. Organizational Party Branch Joint Youth League Branch | Yes |
| 83 | China Salt Heilongjiang Salt Industry Group Invited to Participate in the Heilongjiang Provincial Public Security Department's 2024 National Food Safety Publicity Week Activity "Cracking Down on Food Crimes, Safeguarding the Safety of People's Dining Tables" | 2024 | Industry initiative | Heilongjiang Provincial Public Security Department, Harbin Public Security Bureau | Yes |
| 84 | China Salt Xinjiang Company Carries Out "Healthy Xinjiang Tour" Volunteer Service Activities | 2024 | Industry initiative | Xinjiang Autonomous Region Health Commission | Yes |
| 85 | Healthy China Salt Reduction Action·Integrity Product Community Tour - China Salt Heilongjiang Company Enters Xianglin Mingyuan Community | 2024 | Industry initiative | China National Salt Industry Group Heilongjiang Salt Industry Co., Ltd. | Yes |
| 86 | Healthy China Salt Reduction Action Enters China Southern Airlines Group Beijing Daxing Base | 2024 | Industry initiative | Xinhua News Agency Brand Office | Yes |
| 87 | Zunyi Distribution Center Conducts Low-Sodium Salt Publicity Activities | 2024 | Industry initiative | Guizhou Salt Industry Group Zunyi Distribution Center | Yes |
| 88 | Salt Reduction, Not Saltiness Reduction, Low Sodium for Better Health — Low-Sodium Salt Health Science Lecture Enters Relevant Organizations | 2024 | Industry initiative | Guizhou Salt Industry Group Zunyi Company Suiyang Branch | Yes |
| 89 | Fujian Salt Group Advocates Low-Sodium and Salt Reduction to Jointly Build a Cardiovascular and Cerebrovascular Health Defense Line | 2024 | Industry initiative | Fujian Provincial Health Commission | Yes |
| 90 | Ordos Salt Industry Co., Ltd. Conducts Themed Publicity Campaign "Building a Healthy China, Advocating Consumption of Low-Sodium Salt" | 2024 | Industry initiative | Inner Mongolia Salt Industry Group Chifeng City Salt Industry Co., Ltd. | Yes |
| 91 | Chifeng Salt Industry Co., Ltd. Conducts Low-Sodium Salt Reduction Health Publicity Campaign at Chifeng University | 2024 | Industry initiative | Inner Mongolia Salt Industry Group Chifeng City Salt Industry Co., Ltd. | Yes |
| 92 | Mengyan Group Conducts Public Welfare Science Popularization Activities for "National Hypertension Day" | 2024 | Industry initiative | Inner Mongolia Salt Industry Group Salt Industry Co., Ltd. | Yes |
| 93 | Ordos Salt Industry Co., Ltd. Conducts Party Day Activity "Telling Stories of Ethnic Unity and Progress, Promoting Low-Sodium Salt Products as the Main Theme" | 2024 | Industry initiative | Inner Mongolia Salt Industry Group Erdos City Salt Industry Co., Ltd. | Yes |
| 94 | Hulunbuir Salt Industry Co., Ltd. Collaborates with Hailar District Market Supervision Administration to Conduct "Choose Low Sodium for a Healthy Family" Publicity Campaign | 2024 | Industry initiative | Inner Mongolia Salt Industry Group Hulunbuir City Salt Industry Co., Lt | Yes |
| 95 | Tongliao Salt Industry Co., Ltd. Conducts Themed Publicity Campaign "Low-Sodium Salt Without Reduced Saltiness, Lowering Blood Pressure for Better Health" | 2024 | Industry initiative | Inner Mongolia Salt Industry Group Tongliao City Salt Industry Co., Ltd. | Yes |
| 96 | Salt Reduction Without Reduced Saltiness, Low Sodium for Better Health — Bayannur Salt Industry Company Enters Jiefang Street Yulong Community | 2024 | Industry initiative | Inner Mongolia Salt Industry Group Bayannur City Salt Industry Co., Ltd. | Yes |
| 97 | China Salt Shanghai Company Joins Hands with JD Supermarket to Open a New Chapter of Strategic Cooperation | 2024 | Industry initiative | Zhongyan Shanghai Company | Yes |
| 98 | Healthy China Salt Reduction Action Upgraded Again: China Salt and Xinhua News Agency Jointly Write a New Chapter in "Salt Reduction" | 2024 | Industry initiative | China National Salt Industry Group Co., Ltd | Yes |
| 99 | Municipal Government Notice on Issuing Opinions for Further Implementing the Healthy China Initiative and Building a Model City for Healthy China | 2023 | Policy | Suzhou Municipal People's Government | Yes |
| 100 | Low-Sodium Salt Promotion Exchange Seminar Held in Shanghai | 2023 | Industry initiative | China National Salt Industry Co., Ltd. | Yes |
| 101 | China Salt Brand Low-Sodium Salt Launch and "Low-Sodium Salt into the Community" Activity Kick-off Ceremony Held | 2023 | Industry initiative | Tianjin Food Safety Association | Yes |
| 102 | Yueyan Practices Salt Reduction Action to Guard a Healthy and Beautiful Life | 2023 | Industry initiative | Guangdong Salt Industry Group Shenzhen Co., Ltd. | Yes |
| 103 | Low-Sodium Salt Promotion Action to Cover Over 200 Communities in Beijing | 2022 | Industry initiative | Zhongyan Beijing-Tianjin-Hebei Salt Industry Co., Ltd. | Yes |
| 104 | China Salt Bus Unveiled, Advocating a New Concept of Healthy Low-Sodium Salt Consumption | 2022 | Industry initiative | China National Salt Industry Group Co., Ltd | Yes |
| 105 | Salt Reduction, Not Saltiness Reduction! "Healthy China, Salt Reduction Action" Enters Communities to Popularize Low-Sodium Salt | 2022 | Industry initiative | China National Salt Industry Group Co., Ltd | Yes |
| 106 | China Salt Group Distributes 200 Tons of Low-Sodium Salt to the Public in Yichuan, Shaanxi | 2021 | Industry initiative | China National Salt Industry Group Co., Ltd. | Yes |
| 107 | *Shunyi District Chronic Non-communicable Disease Prevention and Control Work Plan (2014 - 2020)* | 2014 | Action Plan / Strategy Document | Shunyi District People's Government Office | Yes |
| 108 | Wei Wei Participates in "Advocating a Healthy New Lifestyle" Ten-Thousand Signature Event to Promote Low-Sodium Salt | 2014 | Industry initiative | China National Salt Industry Group Heilongjiang Salt Industry Co., Ltd. | Yes |
| 109 | Notice on Issuing the Implementation Plan for Dabu County's "Scientific Salt Use, Promoting Health" Salt Reduction Activity | 2013 | Action Plan / Strategy Document | Dapu County People's Government | Yes |
| 110 | Notice on Issuing the Implementation Plan for Fengshun County's "Scientific Salt Use, Promoting Health" Salt Reduction Activity | 2013 | Action Plan / Strategy Document | Fengshun County People's Government | Yes |
| 111 | Municipal Women's Federation and Municipal Salt Administration Organize Special Training on Women's Health and Scientific Consumption of Low-Sodium Salt | 2012 | Program/Initiative | Ankang Municipal People's Government | Yes |
| 112 | Notice of the Municipal People's Government Office on Effectively Promoting and Popularizing Green Food Iodized Salt and Low-Sodium Salt | 2012 | Policy | Suizhou Municipal People's Government | Yes |
| 113 | China Salt Qingdao Company Conducts "Learning from Lei Feng in Our Positions" Health Publicity Activities | 2012 | Industry initiative | Qingdao Salt Administration | Yes |
| 114 | Eat Less Salt, Eat Good Salt, Prevent Hypertension — Tianjin Low-Sodium Salt Promotion Project Officially Launched | 2011 | Program/Initiative | Tianjin Municipal Health Commission | Yes |
| 115 | Beijing Municipal Commerce Commission Conducts Edible Low-Sodium Salt Promotion Activities | 2010 | Industry initiative | Beijing Municipal Commerce Commission | Yes |

**Table S5 Stakeholder Identification and Coding Framework.**

| **Stakeholder Category** | **Operational definition** | **Coding Rule** | **Example** |
| --- | --- | --- | --- |
| **Government** | The chief administrative authority in China is the State Council. Government agencies are ministries that are under the State Council and centrally administered government organizations that report directly to the State Council. | Government entities are coded according to four administrative levels: national, provincial, municipal, and county/district. If a government body has multiple subordinate agencies with distinct functions, each is recorded as a separate entry. | National Disease Control and Prevention Administration and Chinese Center for Disease Control and Prevention are coded as two separate entities. |
| **Professional associations** | Medical, scientific, and clinical professional associations that develop guidelines, provide expert consensus, and lead professional education | For specialized sub-societies under a larger association, the name of the parent association is uniformly used for coding. | Cardiology Branch of the Chinese Medical Association is coded uniformly as Chinese Medical Association. |
| **Academic institutions** | Academic universities, research institutes, and laboratories conducting public health, clinical, and policy research | Colleges mentioned in documents are coded using the full name of their parent university. Research institutes are coded under their full official names. | School of Public Health, Nanchang University is coded as Nanchang University. |
| **Industry** | Salt and food industry stakeholders involved in production, distribution, sales, and quality management | Local branches affiliated with the China Salt Industry Group are uniformly coded as China Salt Local Branch. Other local salt enterprises not under this group are coded as Other Local Salt Companies. When policy documents refer to the salt industry collectively without specifying company names, the generic code All Salt Companies in China is applied. China Salt Industry Co., Ltd. and China Salt Industry Group Co., Ltd. are coded using their respective official registered names. | China Salt Shanghai Salt Co., Ltd. is coded as China Salt Local Branch. |
| **Non-Governmental Organizations (NGOs)** | International/intergovernmental organizations, non-governmental public health organizations, global health initiatives, and advocacy groups | Organizations are coded using their official names. | Resolve to Save Lives is coded as Resolve to Save Lives. |
| **Media** | Media outlets that shape public opinion on salt substitute and sodium reduction | Satellite television stations operated by local governments are uniformly coded as Local Satellite TV. All other media organizations are coded using their official names. | Inner Mongolia Satellite TV is coded as Local Satellite TV. |
| **Healthcare providers** | Healthcare professionals and clinical institutions involved in diagnosis, treatment, and patient education | All medical institutions are uniformly coded as Healthcare providers. | The First Affiliated Hospital of China Medical University is coded as Healthcare providers. |
| **Public** | Individuals or groups identified as members of the public | Any individuals or groups identified as members of the public are uniformly coded under the single category Public. | All community residents are coded as Public. |

**Table S6 Evidence supporting stakeholder power and interest classification, including documentary sources, coding rationale, uncertainty status, and final decisions after review.**

| Stakeholder Name | Sector | Power classification | Evidence supporting power | Interest classification | Evidence supporting interest | Evidence source | Supporting document ID(s) | Uncertainty status | Final rationale |
| --- | --- | --- | --- | --- | --- | --- | --- | --- | --- |
| Chinese Center for Disease Control and Prevention | Government | High | National public health authority coordinating potassium-enriched low-sodium salt evidence and implementation. | High | Conducts sodium reduction research, surveillance, and implementation activities related to potassium-enriched low-sodium salt. | Technical reports; academic publications; policy documents | 21, 45 | Clear |  |
| National Center for Cardiovascular Diseases | Government | Low | Provides research evidence linking sodium to CVD burden | Low | Few directly related to potassium-enriched low-sodium salt. | Clinical guidelines; academic publications | 21, 51 | Unclear—resolved by discussion | Limited direct evidence of involvement in potassium-enriched low-sodium salt promotion was identified. Classification was discussed among reviewers and resolved as low power and low interest. |
| National Center for Neurological Disorders | Government | Low | Contributes stroke-related evidence | Low | Limited policy authority few directly related to potassium-enriched low-sodium salt. | Clinical guidelines; academic publications | 21 | Unclear—resolved by discussion | Limited evidence of direct involvement in potassium-enriched low-sodium salt-related policy or implementation was identified. Classification was discussed among reviewers and resolved as low power and low interest. |
| National Health Commission | Government | High | Core policymaker and regulator for national salt reduction programs | Low | Few directly related to potassium-enriched low-sodium salt. | Policy documents; official documents; government reports | 20, 21, 23, 29, 30, 39 | Clear |  |
| National Institute for Nutrition and Health, China CDC | Government | High | Leads nutrition surveillance and potassium-enriched low-sodium salt technical guidance. It has strong agenda-setting influence in public health programs, including sodium reduction. | High | Conducts research, technical guidance, and intervention activities related to sodium reduction and potassium-enriched low-sodium salt. | Technical guidelines; academic publications; Clinical guidelines; international reports | 10, 24, 25 | Clear |  |
| National Center for Chronic and Noncommunicable Disease Control and Prevention, China CDC | Government | High | Oversees chronic disease prevention programs and salt reduction initiatives. It has soft power in evidence generation and national health campaigns. | High | Engaged in chronic disease prevention, salt reduction initiatives, and implementation research related to potassium-enriched low-sodium salt. | Technical guidelines; academic publications; Clinical guidelines | 24, 25, 26 | Clear |  |
| State Administration for Market Regulation | Government | High | Regulates food labeling, salt standards, and market compliance | Low | Currently few activities related to potassium-enriched low-sodium salt. | Policy documents; government reports; official documents | 29, 39 | Clear |  |
| Ministry of Industry and Information Technology | Government | High | Oversees industrial policy | Low | Limited documented interest, reflected in minimal engagement in nutrition-related activities. | Policy documents; government reports; official documents | 29, 39 | Clear |  |
| Healthy China Action Promotion Committee | Government | High | Inter-ministerial body ensuring coordination under Healthy China 2030 | Low | Currently few activities related to potassium-enriched low-sodium salt. | Policy documents; government reports; official documents | 44, 45, 66 | Clear |  |
| National Disease Control and Prevention Administration | Government | High | Leads national-level NCD and salt reduction strategy, but currently few activities related to potassium-enriched low-sodium salt. | Low | Leads national-level NCD and salt reduction strategy, but currently few activities related to potassium-enriched low-sodium salt. | Policy documents; government reports; official documents | 29 | Clear |  |
| National Salt Product Quality Supervision and Inspection Center | Government | High | Ensures technical testing and quality control of salt products. | High | Directly involved in the development, technical specification, testing, and quality supervision of low-sodium salt standards and products. | Technical reports; official documents | 41, 42 | Clear |  |
| Health Promotion Department, Chinese Center for Health Education | Government | High | Implements national salt awareness and education campaigns | Low | Few directly related to potassium-enriched low-sodium salt. | Technical guidelines; health promotion reports; academic publications | 9 | Clear |  |
| State Council | Government | High | Sets national health priorities but limited direct engagement in potassium-enriched low-sodium salt. | Low | Sets national health priorities but limited direct engagement in potassium-enriched low-sodium salt. | Policy documents; official documents | 9 | Clear |  |
| National Health and Family Planning Commission | Government | High | Predecessor to NHC | Low | Historical role in policy initiation. | Policy documents; government reports; official documents | 47, 49, 50, 51 | Clear |  |
| National Development and Reform Commission | Government | High | Economic planning authority | Low | Indirect relevance to potassium-enriched low-sodium salt. | Policy documents; government reports; official documents | 29 | Clear |  |
| Provincial Health Commission | Government | High | Implements Healthy China initiatives and regional salt control. | Low | There are very few special initiatives targeting potassium-enriched low-sodium salt, resulting in a low direct relevance. | Policy documents; government reports; official documents | 9, 10, 16, 17, 21, 59, 60, 61, 62, 64, 65, 67, 68, 71, 73, 84, 89, 92, 99, 102, 114 | Clear |  |
| Provincial Disease Control and Prevention Administration | Government | Low | Key provincial technical agency executing potassium-enriched low-sodium salt projects. Technical implementation role without national decision authority | High | Participates in regional disease prevention, surveillance, and implementation of salt reduction activities. | Policy documents; government reports; technical reports | 59, 60, 61, 62, 66 | Unclear—resolved by discussion | Evidence indicated potential involvement in regional disease prevention activities, but direct potassium-enriched low-sodium salt-related engagement was unclear. Classification was discussed among reviewers and resolved as low power and high interest. |
| Provincial Administration for Market Regulation | Government | High | Supervises salt labeling and regional market enforcement. | Low | Limited documented activities specifically related to potassium-enriched low-sodium salt promotion. | Policy documents; government reports; official documents | 59, 61, 62, 65, 67, 68, 99 | Clear |  |
| Provincial People's Government | Government | High | Policy enabler | Low | Engages through directives rather than practice. | Policy documents; government reports; official documents | 68, 69, 70, 74 | Clear |  |
| Provincial Bureau of Quality and Technical Supervision | Government | High | Oversees technical standards | Low | Limited nutrition relevance. | Policy documents; government reports; official documents | 69 | Clear |  |
| Provincial Center for Disease Control and Prevention | Government | Low | Implements surveillance, public education, and potassium-enriched low-sodium salt pilots. | High | Implements surveillance, public education, and potassium-enriched low-sodium salt pilots. | Technical guidelines; health promotion reports; academic publications | 4, 6, 14, 72, 75, 89, 102, 109, 110, 111, 112, 114 | Clear |  |
| Provincial Food and Drug Administration | Government | High | Monitors product safety and salt quality compliance. | Low | Monitors product safety and salt quality compliance. | Policy documents; government reports; official documents | 69 | Clear |  |
| Provincial Development and Reform Commission | Government | High | Economic and industrial planning | Low | Peripheral to potassium-enriched low-sodium salt. | Policy documents; government reports; official documents | 59 | Clear |  |
| Provincial Department of Industry and Information Technology | Government | High | Oversees industry development | Low | Low health orientation. | Policy documents; government reports; official documents | 60, 61, 62, 66, 68 | Clear |  |
| Provincial Department of Economy and Information Technology | Government | High | Focused on industry | Low | Limited link to potassium-enriched low-sodium salt. | Policy documents; government reports; official documents | 69 | Clear |  |
| Provincial Salt Industry Administration | Government | Low | Manages provincial-level salt supply and logistics. Many provinces have abolished this department. | High | Manages provincial-level salt supply and logistics. Many provinces have abolished this department. | Policy documents; government reports; industry documents | 71, 72 | Clear |  |
| Municipal People's Government | Government | High | Local governance | Low | Administrative oversight only. | Policy documents; government reports; official documents | 99, 111, 112 | Clear |  |
| Municipal Administration for Market Regulation | Government | High | Ensures compliance with product and labeling standards. | Low | Ensures compliance with product and labeling standards. | Policy documents; government reports; official documents | 64, 65 | Clear |  |
| Municipal Department of Industry and Information Technology | Government | High | Industry-focused | Low | Minor engagement in potassium-enriched low-sodium salt. | Policy documents; government reports; official documents | 64, 99 | Clear |  |
| Municipal Development and Reform Commission | Government | High | Coordinates economic policy | Low | Low public health role. | Policy documents; government reports; official documents | 64 | Clear |  |
| Municipal Health Commission | Government | High | Local executor of nutrition and salt reduction programs, generally potassium-enriched low-sodium salt is not their priority | Low | Local executor of nutrition and salt reduction programs, generally potassium-enriched low-sodium salt is not their priority | Policy documents; government reports; public health reports | 64, 73, 99, 114 | Clear |  |
| Municipal Salt Industry Administration | Government | Low | It only undertakes auxiliary work concerning the distribution and local rollout of potassium-enriched low-sodium salt | High | Facilitates local potassium-enriched low-sodium salt distribution and enforcement. | Policy documents; government reports; industry documents | 111, 113 | Unclear—resolved by discussion | Responsible for provincial salt supply and distribution management; however, this institution has been abolished in several provinces following administrative reforms. After discussion, it was classified as low power and high interest. |
| Municipal Center for Disease Control and Prevention | Government | Low | It only carries out on-site implementation work such as salt reduction education and data collection at the local level. | High | Executes salt reduction education and data collection locally. | Technical reports; public health reports; program reports | 6, 75, 114 | Clear |  |
| Municipal Department of Economy and Information Technology | Government | High | Industrial affairs | Low | Not central to potassium-enriched low-sodium salt work. | Policy documents; government reports; official documents | 75 | Clear |  |
| Municipal Commerce Commission | Government | High | Oversees trade and retail sectors | Low | Low potassium-enriched low-sodium salt engagement. | Policy documents; government reports; official documents | 115 | Clear |  |
| District/County People's Government | Government | High | Implements higher-level policies | Low | Low strategic input. | Policy documents; government reports; official documents | 107, 109, 110 | Clear |  |
| Chinese Medical Doctor Association | Professional associations | Low | Large membership | Low | Moderate influence on practice behavior. | Clinical guidelines; professional statements; organizational documents | 19, 24, 33, 49, 51 | Clear |  |
| Endocrine Specialty Alliance for the Co-development of Traditional Chinese Medicine in the Beijing-Tianjin-Hebei Region | Professional associations | Low | Specialized TCM alliance | Low | Minimal link to sodium policy. | Clinical guidelines; organizational documents | 19 | Clear |  |
| Beijing Association of Integrated Traditional and Western Medicine | Professional associations | Low | Low engagement and limited health policy influence. | Low | Low engagement and limited health policy influence. | Professional statements; organizational documents | 19 | Clear |  |
| Beijing Hypertension Association | Professional associations | Low | Professional association without regulatory authority but with influence in hypertension management networks. | High | Active in hypertension management and salt awareness advocacy. | Professional statements; organizational documents | 31 | Clear |  |
| Chinese Medical Association | Professional associations | Low | Broad scientific body | Low | Minor direct engagement in potassium-enriched low-sodium salt. | Clinical guidelines; professional statements; organizational documents | 22, 24, 27, 33, 38, 40, 43, 46, 51 | Clear |  |
| Cross-Strait Medical and Health Exchange Association | Professional associations | Low | Limited advocacy | Low | Minimal involvement in salt reduction. | Clinical guidelines; professional statements; organizational documents | 22 | Clear |  |
| Chinese Association of Rehabilitation Medicine | Professional associations | Low | Focused on rehabilitation | Low | No link to sodium policy. | Clinical guidelines; professional statements; organizational documents | 22, 33 | Clear |  |
| Chinese Preventive Medicine Association | Professional associations | Low | Key preventive medicine group promoting NCD risk reduction. | High | Key preventive medicine group promoting NCD risk reduction. | Technical guidelines; professional statements | 24, 43, 46 | Clear |  |
| Chinese Nutrition Society | Professional associations | High | National authority in dietary sodium guidelines and education. | High | Actively promotes sodium reduction and dietary behavior change through nutrition education. | Dietary guidelines; technical guidelines; academic publications | 10, 24, 25, 37, 46 | Clear |  |
| China International Exchange and Promotion Association for Medical and Health Care | Professional associations | Low | Minimal policy role in nutrition reform. | Low | Minimal policy role in nutrition reform. | Clinical guidelines; professional statements; organizational documents | 24, 25, 26, 43, 46 | Clear |  |
| Chinese Hypertension League | Professional associations | Low | Active hypertension network supporting potassium-enriched low-sodium salt promotion. | High | Active hypertension network supporting potassium-enriched low-sodium salt promotion. | Clinical guidelines; professional statements; organizational documents | 24, 25, 26, 51 | Clear |  |
| Chinese Stroke Association | Professional associations | Low | Contributes advocacy linking salt to stroke prevention. | Low | Contributes advocacy linking salt to stroke prevention. | Clinical guidelines; professional statements | 26, 34 | Clear |  |
| Chinese Geriatrics Society | Professional associations | Low | Low engagement | Low | Indirect health relevance. | Clinical guidelines; professional statements | 26, 31 | Clear |  |
| Chinese Geriatric Health Care Association | Professional associations | Low | Minor organization with limited influence. | Low | Minor organization with limited influence. | Professional statements; organizational documents | 26 | Clear |  |
| China National Light Industry Council | Professional associations | Low | Industry group | Low | limited nutrition relevance. | Industry standards; official documents | 41, 52, 53 | Clear |  |
| Chinese Pharmaceutical Association | Professional associations | Low | Focuses on pharmacy affairs | Low | No potassium-enriched low-sodium salt engagement. | Clinical guidelines; professional statements | 28, 47 | Clear |  |
| Chinese Gerontological and Geriatric Medical Society | Professional associations | Low | Focused on aging research | Low | Not policy-active. | Clinical guidelines; professional statements | 33 | Clear |  |
| Chinese Community Health Association | Professional associations | Low | Limited documented interest, reflected in weak engagement in national potassium-enriched low-sodium salt policy. | Low | Limited documented interest, reflected in weak engagement in national potassium-enriched low-sodium salt policy. | Professional statements; organizational documents | 43 | Clear |  |
| Chinese Association of Integrative Medicine | Professional associations | Low | Promotes holistic health | Low | Some engagement in nutrition. | Clinical guidelines; professional statements | 40 | Clear |  |
| Tianjin Food Safety Association | Professional associations | Low | Regional association | Low | Marginal relevance to potassium-enriched low-sodium salt. | Organizational documents; industry documents | 101 | Clear |  |
| Guangdong Provincial Institute of Public Health | Academic institutions | Low | Conducts public health surveillance | Low | Limited policy impact. | Academic publications; technical reports; Clinical guidelines | 42, 100 | Clear |  |
| Chifeng University | Academic institutions | Low | Small academic unit | Low | Minimal relevance. | Industry documents | 91 | Clear |  |
| Ningxia Medical University | Academic institutions | Low | Participates in regional salt reduction trials. | High | Participates in regional salt reduction trials. | Academic publications; research reports | 4, 14, 16, 17 | Clear |  |
| China Medical University | Academic institutions | Low | Conducts potassium-enriched low-sodium salt and public health nutrition research. | High | Conducts potassium-enriched low-sodium salt and public health nutrition research. | Academic publications; research reports | 4, 17 | Clear |  |
| Changzhi Medical College | Academic institutions | Low | Involved in community-based potassium-enriched low-sodium salt interventions. | High | Involved in community-based potassium-enriched low-sodium salt interventions. | Academic publications; research reports | 4, 6, 14, 16, 17 | Clear |  |
| Xi'an Jiaotong University | Academic institutions | Low | Public health teaching institution | Low | Limited engagement. | Academic publications; research reports | 4, 6, 14, 16, 17 | Clear |  |
| Peking University Institute of Clinical Medicine | Academic institutions | Low | National academic leader providing policy-relevant evidence. | High | National academic leader providing policy-relevant evidence. | Academic publications; research reports | 5, 6, 10, 14, 16, 17, 100 | Clear |  |
| Harbin Medical University | Academic institutions | Low | Conducts regional potassium-enriched low-sodium salt pilot research and training. | High | Conducts regional potassium-enriched low-sodium salt pilot research and training. | Academic publications; research reports; trial registrations | 2, 4, 14, 15, 17 | Clear |  |
| Nanchang University | Academic institutions | Low | Contributes applied research and local evaluation studies. | High | Contributes applied research and local evaluation studies. | Academic publications; research reports | 6 | Clear |  |
| Duke Kunshan University | Academic institutions | Low | Collaborates with global potassium-enriched low-sodium salt research partners. | High | Collaborates with global potassium-enriched low-sodium salt research partners. | Academic publications; research reports | 14, 16 | Clear |  |
| The George Institute | Academic institutions | Low | International collaborator generating potassium-enriched low-sodium salt evidence in China. | High | International collaborator generating potassium-enriched low-sodium salt evidence in China. | Academic publications; international reports; policy briefs | 8, 13, 18 | Clear |  |
| National Clinical Research Center for Geriatric Diseases | Academic institutions | Low | Engaged in chronic disease research, not directly potassium-enriched low-sodium salt-focused. | Low | Engaged in chronic disease research, not directly potassium-enriched low-sodium salt-focused. | Clinical guidelines; academic publications; research reports | 31 | Clear |  |
| Chongqing Institute for Food and Drug Control | Academic institutions | Low | Conducts product testing | Low | Peripheral role. | Technical reports; official documents | 42 | Clear |  |
| Hainan Boao Research Center for County-level Medical Development | Academic institutions | Low | Small research unit | Low | Limited engagement. | Clinical guidelines | 28 | Clear |  |
| All Salt Companies in China | Industry | Low | Regional salt producers primarily have commercial interests in potassium-enriched low-sodium salt production and lack administrative authority or regulatory power over salt policy and implementation. | High | Regional manufacturers with growing potassium-enriched low-sodium salt production interest. | Industry documents; media reports; | 44 | Unclear—resolved by discussion | After discussion, regional salt enterprises were classified as low power and high interest because their role is primarily commercial rather than regulatory. Although they contribute to potassium-enriched low-sodium salt production and supply, they lack administrative authority over salt policy and implementation. |
| Other Local Salt Companies | Industry | Low | Regional salt producers primarily have commercial interests in potassium-enriched low-sodium salt production and lack administrative authority or regulatory power over salt policy and implementation. | High | Smaller producers supporting regional supply of potassium-enriched low-sodium salt. | Industry documents; media reports; | 41, 52, 55, 56, 57, 58, 72, 78, 79, 80, 81, 87, 88, 89, 90, 91, 92, 93, 94, 95, 96, 102 | Unclear—resolved by discussion | After discussion, regional salt enterprises were classified as low power and high interest because their role is primarily commercial rather than regulatory. Although they contribute to potassium-enriched low-sodium salt production and supply, they lack administrative authority over salt policy and implementation. |
| Supermarket | Industry | Low | Retail channels | Low | Passive role unless incentivized. | Industry documents; media reports; | 73, 97 | Clear |  |
| Local Branch of China National Salt Industry Corporation | Industry | Low | Regional salt producers primarily have commercial interests in potassium-enriched low-sodium salt production and lack administrative authority or regulatory power over salt policy and implementation. | High | Regional distribution arm supporting potassium-enriched low-sodium salt market expansion. | Industry documents; media reports; | 54, 63, 75, 76, 77, 82, 83, 84, 85, 97, 101, 103, 108, 113, 114, 115 | Unclear—resolved by discussion | After discussion, regional salt enterprises were classified as low power and high interest because their role is primarily commercial rather than regulatory. Although they contribute to potassium-enriched low-sodium salt production and supply, they lack administrative authority over salt policy and implementation. |
| China National Salt Industry Corporation | Industry | High | Primary commercial arm for national potassium-enriched low-sodium salt production. | High | Primary commercial arm for national potassium-enriched low-sodium salt production. | Industry documents; media reports; | 100 | Clear |  |
| China National Salt Industry Group Co., Ltd. | Industry | High | Monopoly actor central to scaling potassium-enriched low-sodium salt nationwide. | High | Monopoly actor central to scaling potassium-enriched low-sodium salt nationwide. | Industry documents; media reports; | 36, 86, 98, 101, 104, 105, 106 | Clear |  |
| Resolve to Save Lives | Non-Governmental Organizations (NGOs) | Low | Provides technical assistance and financial support for sodium reduction. | High | Provides technical assistance and financial support for sodium reduction. | International reports; technical reports; policy briefs | 7, 9, 11, 12 | Clear |  |
| World Health Organization | Non-Governmental Organizations (NGOs) | High | International health organization with global normative influence and technical authority. | Low | Global normative and technical engagement was strong, but direct documented engagement in China-specific potassium-enriched low-sodium salt implementation was limited. | International reports; technical guidelines | 1, 3 | Clear |  |
| Brand Office of Xinhua News Agency | Media | High | Key national media channel supporting public education campaigns. | High | Key national media channel supporting public education campaigns. | Media reports; organizational documents; program reports | 82, 86, 98 | Clear |  |
| Local Satellite TV Station | Media | Low | Local broadcaster with limited health programming focus. | Low | Local broadcaster with limited health programming focus. | Media reports | 92 | Clear |  |
| China Railway Passenger Train Media | Media | Low | Advertising medium | Low | Minimal relevance to potassium-enriched low-sodium salt advocacy. | Media reports; organizational documents | 36 | Clear |  |
| Public | Public | Low | General population as beneficiaries and adopters of potassium-enriched low-sodium salt. | High | General population as beneficiaries and adopters of potassium-enriched low-sodium salt. | Community reports; survey reports | 73, 74, 107, 109, 110, 111, 112, 114 | Clear |  |
| Healthcare providers | Healthcare providers | High | Have direct contact with patients and influence individual dietary decisions. | Low | There is minimal proactive participation and dedicated work specifically targeting potassium-enriched low-sodium salt, leading to low direct relevance. | Clinical guidelines; professional statements | 14, 16, 19, 20, 22, 26, 27, 28, 31, 32, 34, 38, 43, 47, 48, 51, 63, 78, 88, 92, 100 | Clear |  |

Note: Document IDs correspond to Supplementary Table S4; multiple IDs indicate supporting evidence retrieved from distinct source documents. The “Final rationale” column was completed only for stakeholders with unclear classifications to document the basis for the final classification after reviewer discussion and consensus.

**Table S7 Definitions of relationship types.**

| **Relationship Type** | **Description** | **Directional** | **Typical Sources** |
| --- | --- | --- | --- |
| Advocacy | Stakeholder A promotes or endorses work related to potassium-enriched low-sodium salt by Stakeholder B, often through targeted campaigns. | Yes | Press releases, public campaigns, policy reports |
| Collaboration | Stakeholders jointly develop or implement a guideline, study, or program. | No | Project records, guidelines, co-authorship |
| Technical advisory | Stakeholder A offers technical input or serves as an expert to B. | Yes | Expert panels, advisory board listings |
| Policy/regulatory | Stakeholder A issues regulations, policies, or exercises official oversight over Stakeholder B | Yes | Government policy documents, regulations |

**Table S8 Full Names For All Stakeholder Abbreviations**

| **Abbreviation** | **Full Name** | **Stakeholder Category** |
| --- | --- | --- |
| CDC | Chinese Center for Disease Control and Prevention | Government |
| CDC-INH | National Institute for Nutrition and Health, China CDC | Government |
| CDC-NCD | National Center for Chronic and Noncommunicable Disease Control and Prevention, China CDC | Government |
| NSPQSAIC | National Salt Product Quality Supervision and Inspection Center | Government |
| NHC | National Health Commission | Government |
| SAFMR | State Administration for Market Regulation | Government |
| MIIT | Ministry of Industry and Information Technology | Government |
| HCAPC | Healthy China Action Promotion Committee | Government |
| NDCAPA | National Disease Control and Prevention Administration | Government |
| HPCCFHE | Health Promotion Department, Chinese Center for Health Education | Government |
| State Council | State Council | Government |
| NHAFPC | National Health and Family Planning Commission | Government |
| NDARC | National Development and Reform Commission | Government |
| PHC | Provincial Health Commission | Government |
| PAFMR | Provincial Administration for Market Regulation | Government |
| Provincial G | Provincial People’s Government | Government |
| PBOQATS | Provincial Bureau of Quality and Technical Supervision | Government |
| PFADA | Provincial Food and Drug Administration | Government |
| PDARC | Provincial Development and Reform Commission | Government |
| PDOIAIT | Provincial Department of Industry and Information Technology | Government |
| PDOEAIT | Provincial Department of Economy and Information Technology | Government |
| Municipal G | Municipal People’s Government | Government |
| MAFMR | Municipal Administration for Market Regulation | Government |
| MDOIAIT | Municipal Department of Industry and Information Technology | Government |
| MDARC | Municipal Development and Reform Commission | Government |
| MHC | Municipal Health Commission | Government |
| MDOEAIT | Municipal Department of Economy and Information Technology | Government |
| MCC | Municipal Commerce Commission | Government |
| County G | District/County People’s Government | Government |
| NCFCD | National Center for Cardiovascular Diseases | Government |
| NCFND | National Center for Neurological Disorders | Government |
| PDCAPA | Provincial Disease Control and Prevention Administration | Government |
| PCDC | Provincial Center for Disease Control and Prevention | Government |
| PSIA | Provincial Salt Industry Administration | Government |
| MSIA | Municipal Salt Industry Administration | Government |
| MCDC | Municipal Center for Disease Control and Prevention | Government |
| CNS | Chinese Nutrition Society | Professional associations |
| BHA | Beijing Hypertension Association | Professional associations |
| CPMA | Chinese Preventive Medicine Association | Professional associations |
| CHL | Chinese Hypertension League | Professional associations |
| CMDA | Chinese Medical Doctor Association | Professional associations |
| ESAFTCDOTCMITBTHR | Endocrine Specialty Alliance for the Co-development of Traditional Chinese Medicine in the Beijing-Tianjin-Hebei Region | Professional associations |
| BAOITAWM | Beijing Association of Integrated Traditional and Western Medicine | Professional associations |
| CMA | Chinese Medical Association | Professional associations |
| CSMAHEA | Cross-Strait Medical and Health Exchange Association | Professional associations |
| CAORM | Chinese Association of Rehabilitation Medicine | Professional associations |
| CIEAPAFMAHC | China International Exchange and Promotion Association for Medical and Health Care | Professional associations |
| CSA | Chinese Stroke Association | Professional associations |
| CGS | Chinese Geriatrics Society | Professional associations |
| CGHCA | Chinese Geriatric Health Care Association | Professional associations |
| CNLIC | China National Light Industry Council | Professional associations |
| CPA | Chinese Pharmaceutical Association | Professional associations |
| CGAGMS | Chinese Gerontological and Geriatric Medical Society | Professional associations |
| CCHA | Chinese Community Health Association | Professional associations |
| CAOIM | Chinese Association of Integrative Medicine | Professional associations |
| TFSA | Tianjin Food Safety Association | Professional associations |
| NMU | Ningxia Medical University | Academic institutions |
| CMU | China Medical University | Academic institutions |
| CMC | Changzhi Medical College | Academic institutions |
| PUIOCM | Peking University Institute of Clinical Medicine | Academic institutions |
| HMU | Harbin Medical University | Academic institutions |
| NCU | Nanchang University | Academic institutions |
| DKU | Duke Kunshan University | Academic institutions |
| TGI | The George Institute | Academic institutions |
| GPIOPH | Guangdong Provincial Institute of Public Health | Academic institutions |
| Chifeng Univ | Chifeng University | Academic institutions |
| XJTU | Xi'an Jiaotong University | Academic institutions |
| NCRCFGD | National Clinical Research Center for Geriatric Diseases | Academic institutions |
| CIFFADC | Chongqing Institute for Food and Drug Control | Academic institutions |
| HBRCFCLMD | Hainan Boao Research Center for County-level Medical Development | Academic institutions |
| ASCIC | All Salt Companies in China | Industry |
| OLSC | Other Local Salt Companies | Industry |
| LB China Salt Group | Local Branch of China National Salt Industry Corporation | Industry |
| Supermarket | Supermarket | Industry |
| CNSIC Ltd. | China National Salt Industry Corporation | Industry |
| China Salt Group | China National Salt Industry Group Co., Ltd. | Industry |
| WHO | World Health Organization | Non-Governmental Organizations (NGOs) |
| RTSL | Resolve to Save Lives | Non-Governmental Organizations (NGOs) |
| Xinhua Brand | Brand Office of Xinhua News Agency | Media |
| LSTV | Local Satellite TV Station | Media |
| CRPTM | China Railway Passenger Train Media | Media |
| HCPs | Healthcare providers | Healthcare providers |
| Public | General public | Public |

**Table S9 Documentary evidence supporting the classification and network positions of the eight Key Players**

| Stakeholder | Stakeholder category | Power–interest classification | Representative documentary evidence | Rationale for classification | Network position and centrality |
| --- | --- | --- | --- | --- | --- |
| Chinese Center for Disease Control and Prevention | Government | Key Player | 2024 National Hypertension Day: Healthy Weight, Ideal Blood Pressure | Provided technical support, health promotion guidance, and implementation support for salt-reduction activities. | Policy/regulatory: in-degree = 2, out-degree = 0, betweenness = 0; Technical advisory: in-degree = 1, out-degree = 0, betweenness = 0; Collaboration: degree = 1, betweenness = 0 |
| National Institute for Nutrition and Health, China CDC | Government | Key Player | Chinese Guidelines for the Promotion and Application of Low-Sodium Salt (2024) | Provided scientific evidence and technical recommendations related to dietary sodium reduction and low-sodium salt application. | Technical advisory: in-degree = 1, out-degree = 0, betweenness = 0; Collaboration: degree = 9, betweenness = 92.4 |
| National Center for Chronic and Noncommunicable Disease Control and Prevention, China CDC | Government | Key Player | Chinese Guidelines for the Promotion and Application of Low-Sodium Salt (2024) | Supported chronic disease prevention programs and contributed to evidence-based implementation strategies for salt reduction. | Advocacy: in-degree = 1, out-degree = 0, betweenness = 0; Technical advisory: in-degree = 2, out-degree = 0, betweenness = 0; Collaboration: degree = 11, betweenness = 173.1 |
| National Salt Product Quality Supervision and Inspection Center | Government | Key Player | Light Industry Standard of the People’s Republic of China QB/T 2019–2020 Low Sodium Salt | Responsible for technical standards, product specifications, and quality supervision related to low-sodium salt products. | Technical advisory: in-degree = 0, out-degree = 1, betweenness = 0; Collaboration: degree = 4, betweenness = 104.0 |
| Chinese Nutrition Society | Professional associations | Key Player | Chinese Food Industry Salt Reduction Guideline (2024) | National authority in dietary sodium guidelines and education, with active engagement in sodium reduction and dietary behavior change through nutrition education. | Technical advisory: in-degree = 1, out-degree = 0, betweenness = 0; Collaboration: degree = 8, betweenness = 45.7 |
| China National Salt Industry Corporation | Industry | Key Player | Low-Sodium Salt Promotion Exchange Seminar Held in Shanghai (2023) | Primary commercial actor involved in national potassium-enriched low-sodium salt production, reflecting substantial market influence and direct interest in low-sodium salt production and promotion. | Technical advisory: in-degree = 3, out-degree = 0, betweenness = 0 |
| China National Salt Industry Group Co., Ltd. | Industry | Key Player | China Salt Brand Low-Sodium Salt Launch and “Low-Sodium Salt into the Community” Activity Kick-off Ceremony Held (2023) | Major producer and promoter of low-sodium salt products, involved in product development, distribution, and public promotion activities. | Advocacy: in-degree = 0, out-degree = 1, betweenness = 0; Technical advisory: in-degree = 0, out-degree = 1, betweenness = 0; Collaboration: degree = 6, betweenness = 138.4 |
| Brand Office of Xinhua News Agency | Media | Key Player | Healthy China Salt Reduction Action Enters China Southern Airlines Group Beijing Daxing Base (2024) | Key national media actor supporting public education and promotion activities, with substantial communication influence and direct engagement in salt-reduction campaigns. | Advocacy: in-degree = 0, out-degree = 1, betweenness = 0; Collaboration: degree = 1, betweenness = 0 |

Note: The table presents all eight stakeholders classified as Key Players (high power and high interest) in the power–interest matrix. Representative documentary evidence and classification rationales are provided to illustrate the basis for stakeholder classification. Network positions and centrality measures are reported for the relationship-specific networks in which each stakeholder participated.

**Supplementary Figure S1.**

**Supplementary Figure S2.**

Enlarged stakeholder network diagrams. Enlarged views of the advocacy, collaboration, technical advisory, and policy/regulatory networks are provided to improve readability of stakeholder labels. Stakeholder abbreviations are used for visualization purposes, and full names are provided in Supplementary Table S8.

(A) Policy/regulatory network

(B) Technical advisory network

(C) Collaboration network

(D) Advocacy network
